# Population-based prevalence and mutational landscape of von Willebrand disease using large-scale genetic databases

**DOI:** 10.1038/s41525-023-00375-8

**Published:** 2023-10-16

**Authors:** Omid Seidizadeh, Andrea Cairo, Luciano Baronciani, Luca Valenti, Flora Peyvandi

**Affiliations:** 1 Fondazione IRCCS Ca’Granda Ospedale Maggiore Policlinico, Angelo Bianchi Bonomi Hemophilia and Thrombosis Center, Milan, Italy; 2 Department of Pathophysiology and Transplantation, Università degli Studi di Milano, Milan, Italy; 3 Fondazione IRCCS Ca’Granda Ospedale Maggiore Policlinico, Precision Medicine Lab, Biological Resource Center, Department of Transfusion Medicine, Milan, Italy

**Keywords:** Haematological diseases, Medical genetics

## Abstract

Von Willebrand disease (VWD) is a common bleeding disorder caused by mutations in the von Willebrand factor gene (*VWF*). The true global prevalence of VWD has not been accurately established. We estimated the worldwide and within-population prevalence of inherited VWD by analyzing exome and genome data of 141,456 individuals gathered by the genome Aggregation Database (gnomAD). We also extended our data deepening by mining the main databases containing *VWF* variants i.e., the Leiden Open Variation Database (LOVD) and the Human Gene Mutation Database (HGMD) with the goal to explore the global mutational spectrum of VWD. A total of 4,313 *VWF* variants were identified in the gnomAD population, of which 505 were predicted to be pathogenic or already reported to be associated with VWD. Among the 282,912 alleles analyzed, 31,785 were affected by the aforementioned variants. The global prevalence of dominant VWD in 1000 individuals was established to be 74 for type 1, 3 for 2A, 3 for 2B and 6 for 2M. The global prevalences for recessive VWD forms (type 2N and type 3) were 0.31 and 0.7 in 1000 individuals, respectively. This comprehensive analysis provided a global mutational landscape of *VWF* by means of 927 already reported variants in the HGMD and LOVD datasets and 287 novel pathogenic variants identified in the gnomAD. Our results reveal that there is a considerably higher than expected prevalence of putative disease alleles and variants associated with VWD and suggest that a large number of VWD patients are undiagnosed.

## Introduction

von Willebrand factor (VWF) is a large glycoprotein synthesized exclusively by endothelial cells (ECs) and megakaryocytes^1^. In order to form a fully functional protein with high-molecular-weight multimers (HMWM), VWF undergoes a sequence of posttranslational modifications including dimerization, multimerization, N- and O glycosylation, sialylation, and sulfation, before being secreted into the circulation^2^. Biosynthesis of VWF begins with a 2813-amino acid (aa) pre-promonomer, composed of a 22 aa signal peptide, a 741 aa pro-peptide (VWFpp), and the mature VWF with 2050 aa. The pro-VWF monomer is a glycoprotein composed of repetitive domain sequences: D1-D2 (VWFpp) -D’-D3-A1-A2-A3-D4-C1-C2-C3-C4-C5-C6-CK (mature subunit)^3^. VWF through its A1 domain binds to the platelet glycoprotein (GP) Iba and collagens IV and VI, through the A3 domain to collagens I and III, and through the D’-D3 domains to coagulation factor VIII (FVIII)^2^. Therefore, VWF plays a key role in both primary (platelet-mediated) and secondary hemostasis (coagulation-mediated)^4,5^.

The VWF gene (*VWF*) was cloned and sequenced in 1985^6–9^. The large gene contains 178 kb of genomic DNA, including 52 exons ranging in size from 40 to 1379 bases, and is located on the short arm of chromosome 12 (12p13.2)^6–9^. A partial *VWF* pseudogene (VWFP) is present in chromosome 22q11.2, spans 25 kb, and has 97% sequence homology with exons 23–34 of *VWF*^10^. The transcriptionally expressed mRNA of VWF is approximately 8.7 kb in length.

Because VWF is essential for normal hemostasis, a deficiency or dysfunction of VWF leads to the common bleeding disorder, von Willebrand disease (VWD). The quantitative defect of VWF can be partial or complete leading to type 1 or type 3 VWD. Qualitative defects result in four different VWD types 2 (2A, 2B, 2M, and 2N)^11^. The genetic variants responsible for type 1 (mostly dominant) and 3 VWD (recessive) are spread across the 52 exons of *VWF*^12–14^, whereas type 2 VWD variants are confined to VWF functional domains^12,15^.

According to previous studies, VWD prevalence is estimated to vary between 0.6% and 1.3%^16,17^, even though on the basis of cases referred to specialized centers about 1 case per 1000 is estimated to have clinically relevant VWD^18,19^. This notwithstanding, the true prevalence of VWD has not been accurately established due to a lack of prospective and systematic studies and to the fact that some patients with *VWF* variants are asymptomatic or have mild clinical manifestations. In addition, the number of people investigated in the aforementioned studies was not large enough to estimate global VWD prevalence and these studies were limited to a small number of geographic areas. A growing number of large-scale population-based sequencing studies are being conducted using massively parallel sequencing, next-generation sequencing (NGS). By using genetic data and specialized statistical techniques, the estimate of disease prevalence can be obtained by means of allele frequency information from a large number of sequenced samples. With this background and gaps of knowledge, we chose to examine the global mutational landscape of *VWF* and to assess the worldwide and within-population prevalence of inherited VWD by analyzing exome and genome data of more than 141,000 individuals gathered by the genome Aggregation Database (gnomAD). We further extended and deepened data mining to the two primary databases containing *VWF* variants, i.e., the Leiden Open Variation Database (LOVD) and the Human Gene Mutation Database (HGMD) with the goal to analyze the global mutational spectrum of VWD.

## Results

### Global mutational spectrum of the *VWF* using population-based exome and genome sequencing data

We collected high-quality data from gnomAD including 141,456 subjects with different ethnicities (Table 1), i.e., Africans/African Americans (12,487 subjects), Latinos/Admixed Americans (17,720), Ashkenazi Jews (5,185), East Asians (9,977), Finnish Europeans (12,562), non-Finnish Europeans (64,603), South Asians (15,308) and also 3,614 additional persons without an assigned ethnicity. The gender distribution of participants was 54% males and 46% females.

**Table 1:** GnomAD database composition according to population details.

Population	Exomes	Genomes	Total
African/African American	8128	4359	12,487
Latino/Admixed American	17,296	424	17,720
Ashkenazi Jewish	5040	145	5185
East Asian	9197	780	9977
European (Finnish)	10,824	1738	12,562
European (non-Finnish)	56,885	7718	64,603
South Asian	15,308	–	15,308
Other	3070	544	3614
Total	125,748	15,708	141,456
XX	57,787	6967	64,754
XY	67,961	8741	76,702

**Fig. 1:**
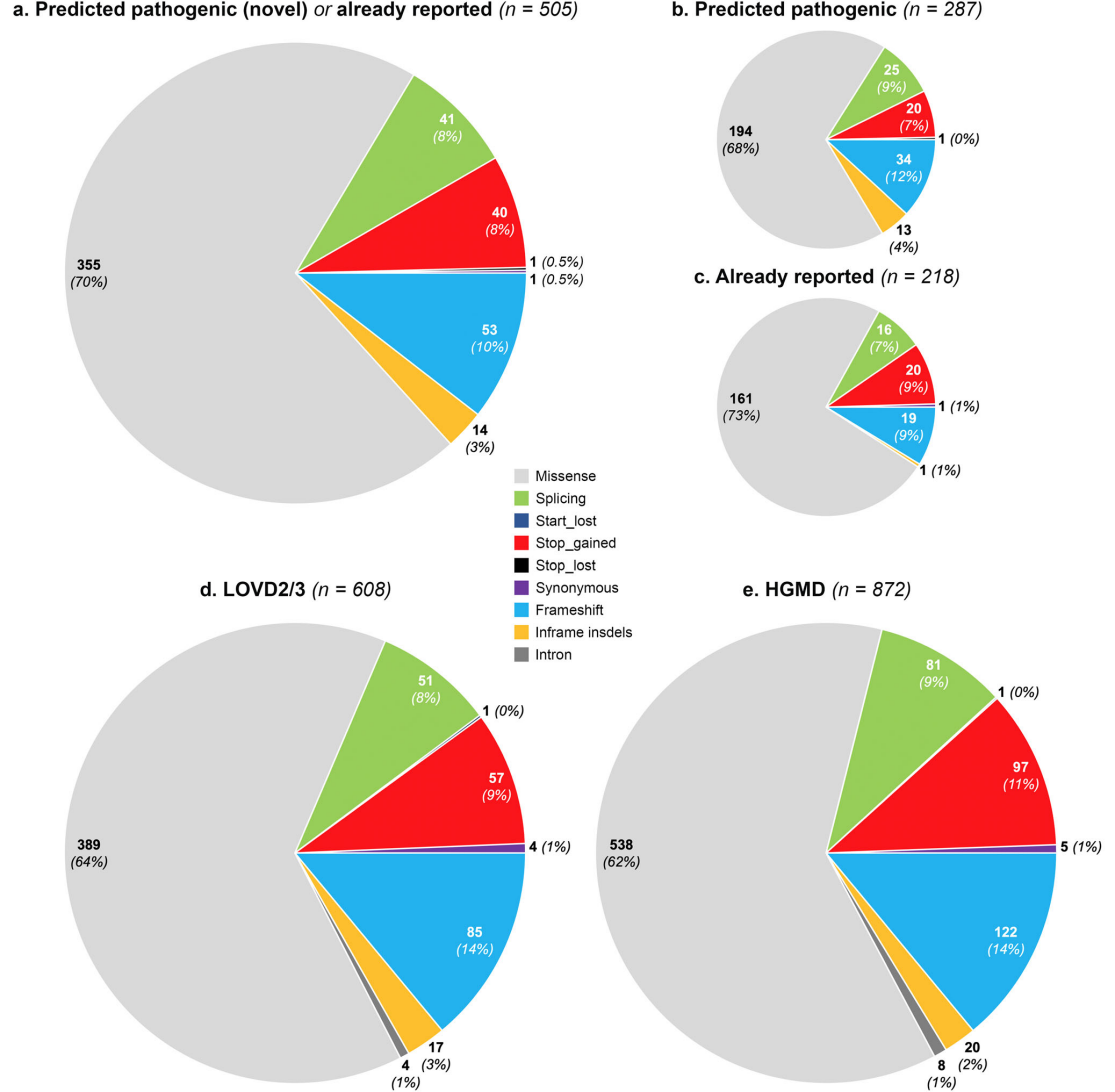
Distribution of various mutation types for *VWF* genetic variants identified in the gnomAD, HGMD and LOVD databases. **a** Identified pathogenic variants in the gnomAD population (*n* = 505) including novel predicted pathogenic variants (*n* = 287, **b**), and those already being reported to be associated with VWD (*n* = 218, **c**). **d**, **e** *VWF* variants (*n* = 927) that have been reported so far to be associated with VWD in LOVD (**d**) and HGMD (**e**).

The mean depth of coverage per base in all *VWF* exons was generally greater than 30 for both exome and genome sequencing except for exon 26 (Supplementary Fig. 1). The lower coverage of exon 26 is primarily due to alignment of the sequences with human genome reference, being aligned with the pseudogene instead of the *VWF*. Since the minimum depth of coverage of gnomAD is set at 10 (DP > = 10), only genotypes that pass this threshold were included in our study, and exon 26 has a depth of coverage higher than this threshold. A total of 4,313 different genetic variants were identified within *VWF* in the gnomAD population. Following a conservative approach to classify variants as pathogenic (i.e., as responsible for VWD), we found 505 distinct *VWF* deleterious variants of which 287 (57%) have not been reported to be associated with VWD in the literature nor in VWD-related databases (Supplementary Table 1), whereas 218 (43%) had been already reported (Supplementary Table 2). The distribution of mutation types for 505 variants identified in the gnomAD is depicted in Fig. 1. Missense accounted for the majority of variants (*n* = 355, 70%) followed by frameshift (*n* = 53, 10%). Gene variants affecting stop codons including stop-gained (*n* = 40, 8%) and stop-loss (*n* = 1) as well as variants affecting a splicing site (*n* = 41, 8%) were also identified. There were also 14 inframe insdels (3%) and one synonymous variant (Fig. 1a). A similar distribution of mutation types was observed between novel (*n* = 287) and previously reported (*n* = 218) variants (Fig. 1a). Data on gene constraint provided by the gnomAD indicates that *VWF* seems to be intolerant to missense variants while being tolerant of synonymous and loss-of-function variants (Supplementary Table 3).

Out of the 505 selected pathogenic variants, 244 (48%) were unique and each variant was identified in one subject only. The frequency of novel variants (*n* = 287) was much higher in non-Finnish Europeans (35%), Africans/African Americans (27%), Latinos/Admixed Americans (18%) and to a lesser rate in East Asians (13%). However, only 3% were identified in Ashkenazi Jews and 2% in Finnish Europeans. Among a total number of 282,912 alleles analyzed, 31,785 contained *VWF* pathogenic variants. Only 2.9% of the affected alleles were carrying of the novel variants. In the East Asian population, as many as 18.9% of affected alleles carried novel variants, whereas among other ethnicities the impact of novel variants was considerably lower (1.3–4%, Table 2).

**Table 2:** The number of affected alleles by already reported and novel variants identified in the gnomAD population.

Population	Total number of affected alleles	Total number of alleles affected by reported variants	Total number of affected alleles by novel variants	% of alleles affected by novel variants
All	31,785	30,850	935	2.9
African/African American	14,458	14,236	222	1.5
Latino/Admixed American	3673	3530	143	3.9
Ashkenazi Jewish	846	820	26	3.1
East Asian	546	443	103	18.9
Finnish	1238	1222	16	1.3
European (not Finnish)	7573	7282	291	3.8
South Asian	2840	2725	115	4.0
Other ethnicities	611	592	19	3.1

Among the 141,456 participants in the gnomAD, 1206 were homozygotes for 26 different *VWF* pathogenic variants (Supplementary Table 4), the rest of those with pathogenic variants being heterozygotes or compound heterozygotes.

### Mutational spectrum of the *VWF* in the HGMD and LOVD databases

When data analysis was extended to the two main databases containing VWD-associated variants, i.e., HGMD and LOVD, we found that 1024 different *VWF* variants have been so far associated with VWD, 927 of them being single nucleotide variant (SNV) and short insertions/deletions. Of the latter variants, 872 were found in HGMD and 608 in LOVD. Our findings show that the distribution of *VWF* mutation types in the gnomAD dataset was similar to those in the HGMD and LOVD and did not change between the novel and already reported variants (Fig. 1).

### VWD type distribution in the gnomAD population and HGMD/LOVD datasets

**Fig. 2:**
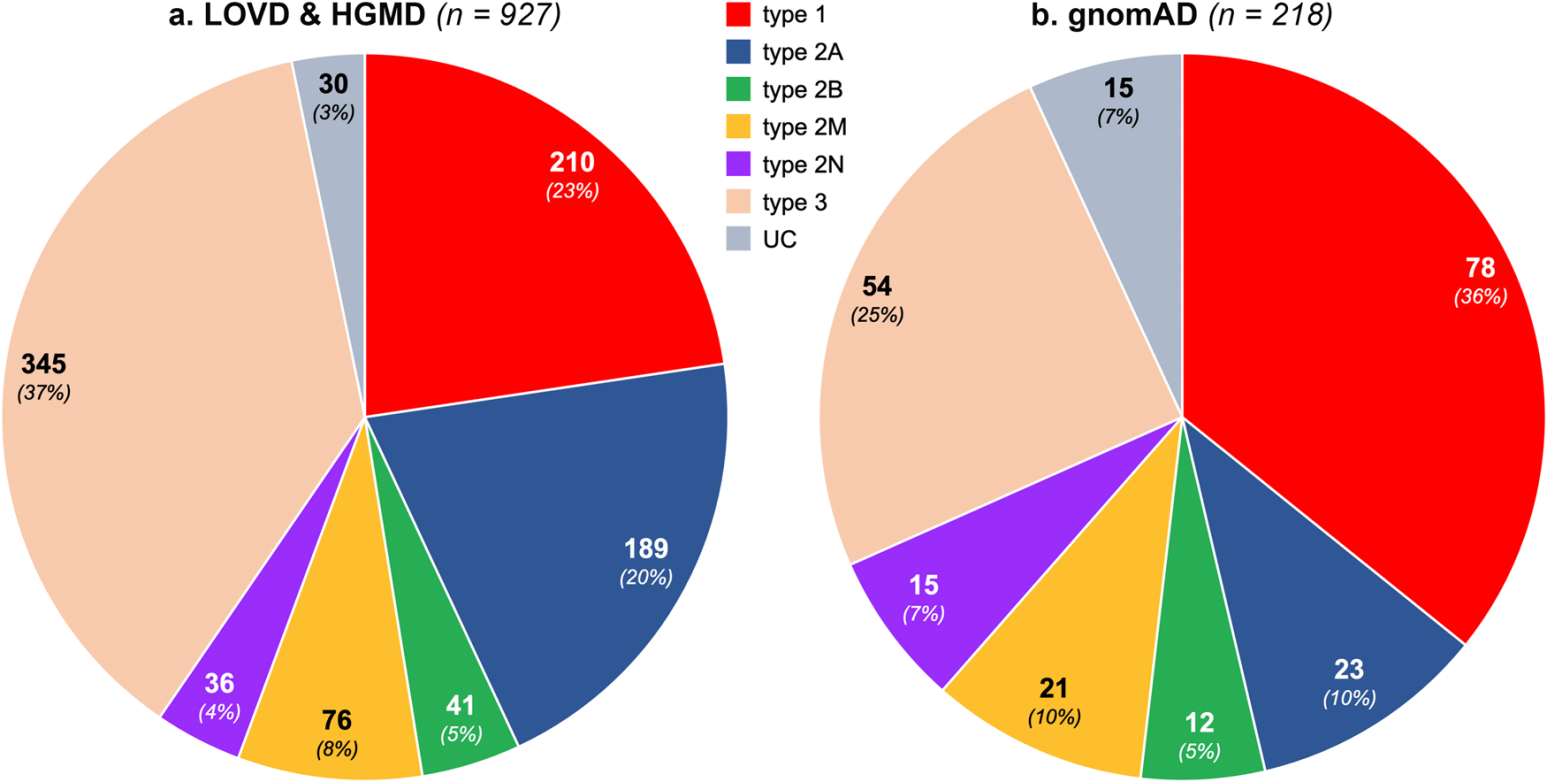
VWD type distribution of all the so far reported and gnomAD identified variants. **a** According to our analysis 927 *VWF* variants (SNV and short insertions/deletions) have been reported so far in the two VWD-related databases (HGMD and/or LOVD) to be associated with VWD. Of which, 555 (60%) were reported in quantitative VWF defects including type 3 VWD (*n* = 345, 37%) and type 1 (*n* = 210, 23%). For type 2 VWD with qualitative VWF defects, 20% were reported in type 2A (*n* = 189), 8% in type 2M (*n* = 76), 5% in type 2B (*n* = 41) and 4% in type 2N (*n* = 36). Out of 4313 different *VWF* variants, we identified 505 pathogenic variants in the gnomAD population of which 287 were novel and 218 were already reported in patients with VWD. **b** For the latter group, the number of *VWF* variants identified was higher for type 1 (*n* = 78, 36%) than type 3 VWD (*n* = 54, 25%). Among type 2 variants identified in the gnomAD, 10% (*n* = 23) were type 2A, 10% (*n* = 21) 2M, 7% (*n* = 15) 2N and 5% type 2B (*n* = 12).

In the gnomAD population, 218 of 505 different pathogenic variants have been already reported to be associated with VWD, of which 61% were responsible for quantitative VWF defects, including 36% for type 1 and 25% for type 3. For qualitative defects, 10% were type 2A, 10% type 2M, 7% type 2N, and 5% type 2B. About 7% of these identified variants were unclassified (UCs). Comparing these data with the so far reported variants in VWD, a higher proportion of genetic variants of type 1, 2M, 2N and UCs were found in the gnomAD population (Fig. 2).

### Domain distribution of *VWF* variants in the gnomAD, HGMD and LOVD datasets

**Fig. 3:**
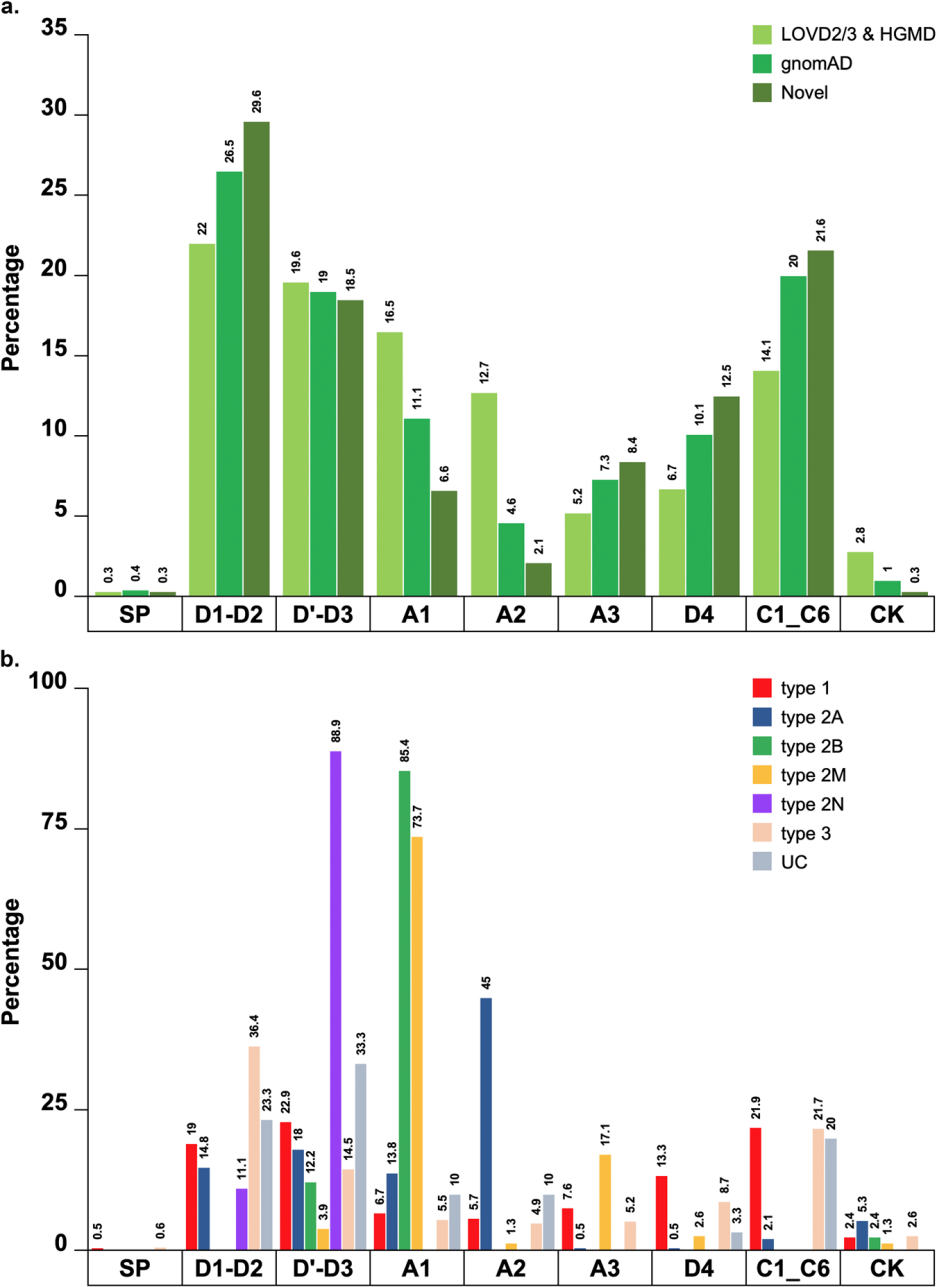
VWF domain distribution and the type of VWD for all the so-far reported (SNV and short insertions/deletions) and pathogenic variants selected from gnomAD. **a** There were fewer variants in the D’-D3, A1, A2 and CK domains among all identified (*n* = 505) and novel variants (*n* = 287) in the gnomAD population compared with those of HGMD and LOVD datasets. **b** We further explored the location of different VWD types on VWF domains for all the so-far reported (SNV and short insertions/deletions) variants in the HGMD and LOVD datasets. Variants of type 1 and 3 VWD were found all over the VWF domains, mainly VWFpp (D1-D2 domain), D’-D3, D4 and C1-C6. In type 2A, 45% of variants were at the A2 domain and the rest were at the D1-D2 (15%), D’-D3 (18%), A1 (14%) and CK domain (5%). All variants of type 2B were at the A1 domain (85%) or D3-A1 junction (12%). Type 2M variants were located mostly at the A1 (74%) but also A3 domains (17%) with a few exceptions on the other domains. A majority of type 2N variants were at the D’-D3 (89%) and the rest 11% at the VWFpp. The unclassified *VWF* variants (UC) were distributed throughout the VWF domains.

The domain distribution of all *VWF* variants in the HGMD and LOVD datasets, all variants selected in the gnomAD (*n* = 505) and the novel variants in this database (*n* = 287) are shown in Fig. 3. Among all pathogenic variants in gnomAD and also among those novel, fewer variants were identified in the VWF D’-D3, A1-A2, and CK domains. However, more novel variants were identified in the D1-D2, A3, D4 and C1-C6 domains (Fig. 3). We further explored the location on VWF domains of different VWD types for all variants in the HGMD and LOVD datasets (Fig. 3). Type 1 and 3 VWD variants were spread all over the VWF domains, mostly at D1-D2, D’-D3 and C1-C6. For type 2A VWD, 45% of variants were located at the A2 domain and the rest at D1-D2 (15%), D’-D3 (18%), A1 (14%) and CK domains (5%). All variants of type 2B VWD were located at the A1 domain (85%) or D3-A1 junction (12%). Almost all type 2M variants were at the A1 (74%) or A3 domains (17%), with a few exceptions at the other remaining domains. The majority of type 2N variants were at the D’-D3 (89%), and the rest 11% at the VWFpp. The UCs were distributed throughout all domains.

### Most frequent variants in the gnomAD population stratified by VWD type and ethnicity

The five most frequent *VWF* variants identified in each ethnic group are shown in Table 3. Several *VWF* variants previously associated with VWF deficiency were relatively common in different ethnicities: p.Arg2185Gln, p.Met740Ile, p.Pro2063Ser, p.His817Gln, p.Arg924Gln, p.Met576Ile, p.Thr2647Met, p.Gly967Asp, p.Thr1034del and p.Ser1731Thr. Generally, in all ethnicities type 1 variants were the most frequent (Table 3). Two type 2N variants were recurrent in Africans/African Americans (p.His817Gln, MAF = 0.115), Latinos/Admixed Americans (p.His817Gln, MAF = 0.0062), Finnish (p.Arg854Gln, MAF = 0.0056) and non-Finnish Europeans (p.Arg854Gln, MAF = 0.0053). For type 2M, p.Ser1731Thr was common in Ashkenazi Jews (MAF = 0.0209) and p.Val1439Met was one of the most frequent variants in Finnish Europeans (MAF = 0.0048). The type 2A variants p.Gly624Ser (MAF = 0.0048) and p.Gly1672Arg (MAF = 0.0018) were among the most frequent variants in East Asians. Type 2B variants, including p.Pro1266Leu (MAF = 0.0036) and p.Asn1231Ser (MAF = 0.0099) were among the most frequent in Finnish Europeans and South Asians. Also type 3 VWD variants were identified in Africans/African Americans (p.Thr1034del, MAF = 0.0152) and South Asians (c.1730-5 C > T, MAF = 0.0049).

**Table 3:** Most frequent ethnicity-specific variants identified in gnomAD with an already established association with VWF deficiency.

Ethnic group	c.DNA	Protein	rs ID	Type of variant	MAF	VWD type of variant
African/African American	c.6554 G > A	p.Arg2185Gln	rs2229446	missense	0.189923	type 1
	c.2220 G > A	p.Met740Ile	rs2228317	missense	0.180051	UC
	c.2451 T > A	p.His817Gln	rs57950734	missense	0.115702	type 2 N
	c.2900 G > A	p.Gly967Asp	rs141087261	missense	0.025651	UC
	c.3101_3103delCCA	p.Thr1034del	rs368366214	inframe_deletion	0.015222	type 3
Latino/Admixed American	c.1728G>T	p.Met576Ile	rs150146744	missense	0.037514	type 1
	c.2220 G > A	p.Met740Ile	rs2228317	missense	0.011653	UC
	c.6554 G > A	p.Arg2185Gln	rs2229446	missense	0.010617	type 1
	c.6187 C > T	p.Pro2063Ser	rs61750615	missense	0.010243	type 1
	c.2451 T > A	p.His817Gln	rs57950734	missense	0.006209	type 2 N
Ashkenazi Jewish	c.6187 C > T	p.Pro2063Ser	rs61750615	missense	0.024976	type 1
	c.5191 T > A	p.Ser1731Thr	rs61750603	missense	0.020926	type 2 M
	c.7025 G > A	p.Arg2342His	rs34120165	missense	0.006853	UC
	c.6554 G > A	p.Arg2185Gln	rs2229446	missense	0.005032	type 1
	c.2220 G > A	p.Met740Ile	rs2228317	missense	0.004341	UC
East Asian	c.6104 G > A	p.Gly2035Asp	rs186806674	missense	0.005613	type 1
	c.6860 G > A	p.Arg2287Gln	rs563856279	missense	0.005415	type 1
	c.1870G>A	p.Gly624Ser	rs542226383	missense	0.004886	type 2 A
	c.2967+2 T > C	/	rs773737583	splice	0.001905	novel
	c.5014 G > A	p.Gly1672 Arg	rs61750598	missense	0.00186	type 2A
Finnish	c.7940 C > T	p.Thr2647Met	rs61751302	missense	0.019783	type 1
	c.2561 G > A	p.Arg854Gln	rs41276738	missense	0.005692	type 2 N
	c.2771 G > A	p.Arg924Gln	rs33978901	missense	0.005573	type 1
	c.4315 G > A	p.Val1439Met	rs150077670	missense	0.004857	type 2 M
	c.3797 C > T	p.Pro1266Leu	rs61749370	missense	0.003667	type 2B
European	c.2771 G > A	p.Arg924Gln	rs33978901	missense	0.018664	type 1
	c.6187 C > T	p.Pro2063Ser	rs61750615	missense	0.008061	type 1
	c.2561 G > A	p.Arg854Gln	rs41276738	missense	0.005343	type 2 N
	c.4751 A > G	p.Tyr1584Cys	rs1800386	missense	0.004024	type 1
	c.7940 C > T	p.Thr2647Met	rs61751302	missense	0.003701	type 1
South Asian	c.6187 C > T	p.Pro2063Ser	rs61750615	missense	0.048537	type 1
	c.3692 A > G	p.Asn1231Ser	rs61749368	missense	0.009904	type 2B
	c.1730-5 C > T	/	rs569984866	splice	0.004989	type 3
	c.6554 G > A	p.Arg2185Gln	rs2229446	missense	0.004313	type 1
	c.2771 G > A	p.Arg924Gln	rs33978901	missense	0.003658	type 1

Ten different variants had a MAF > 0.01 (1%) in at least one population (Supplementary Table 4). Of them, five had an overall population MAF of >1% in Africans/African Americans (p.Arg2185Gln, p.Met740Ile and p.His817Gln), Latinos/Admixed Americans (p.Arg2185Gln, p.Met740Ile and p.Pro2063Ser), Ashkenazi Jews (p.Pro2063Ser), non-Finnish Europeans (p.Arg924Gln) and South Asians (p.Pro2063Ser). Linkage disequilibrium analysis revealed that the three more common variants in Africans/African Americans (p.Arg2185Gln, p.Met740Ile and p.His817Gln) did cosegregate within a common haplotype in 8% of the 1000 genomes project (Supplementary Fig. 2), whereas no combination of these 3 or even 2 variants were observed in other ethnicities.

### Population-based prevalence of autosomal recessive- and dominant VWD

We calculated the worldwide and within population prevalence of VWD for both autosomal dominant and recessive forms, because VWD can be inherited in both patterns (type 1, 2A, 2B and 2M as dominant, type 3 and 2N as recessive). When we considered all identified pathogenic variants (*n* = 505), 13% of the gnomAD alleles carried *VWF* pathogenic variants in the heterozygous state and 0.48% in the recessive state (Table 4). The aforementioned overall frequency estimation was calculated after removing the 3 common variants in African/Americans (p.Arg2185Gln, p.Met740Ile and p.His817Gln). In African/American the frequencies of carriership and recessive forms were 17.2% and 0.90%, respectively. A similar estimated frequency was found for Latino/Admixed Americans (18.6% and 1.07%), South Asians (16.8% and 0.86%) and Ashkenazi Jews (15% and 0.67%), whereas a lower prevalence was estimated among East Asians (5.3% and 0.07%), Finnish (9.4% and 0.24%) and non-Finnish Europeans (11% and 0.34%). In the second approach meant to estimate the global prevalence of *VWF* alleles with pathogenic variants, analysis was limited only to the identified gnomAD variants previously described in VWD (n = 218). The analysis showed an estimation almost identical to the former approach (Table 4), indicating that the novel variants identified are very rare. Indeed, the novel variants identified in the gnomAD affected only about 3% of mutant *VWF* alleles (935 alleles of 31785, Table 2). To calculate the true global prevalence of dominant and recessive VWD types, we used only the variants reported to be associated with VWD in the gnomAD population (n = 218) with an already established autosomal dominant or recessive inheritance pattern. The global prevalence of dominant VWD was 7.4% for type 1, 0.3% for 2A, 0.3% for 2B and 0.6% for 2M. For the recessive VWD forms, it was 0.31% for 2N and 0.7% for type 3 (Table 5). The within-population prevalence of VWD subtypes is summarized in Table 5 and Supplementary Tables 5–10.

**Table 4:** Estimated global prevalence of carriership and recessive *VWF* variants.

Population	Total number of alleles	Total Number of Alleles	Collective frequency of affected alleles	Heterozygote frequency	Prevalence in 100 individuals (autosomal recessive) using all variants (*n* = 505)	Prevalence in 100 individuals (autosomal dominant) using all variants (*n* = 505)	Prevalence in 100 individuals (autosomal recessive) using reported variants (*n* = 218)	Prevalence in 100 individuals (autosomal dominant) using reported variants (*n* = 218)
All^a^	282,912	19,693	0.07	0.14	0.48	13	0.44	12.4
Latino/Admixed American	35,440	3673	0.10	0.21	1.07	18.6	0.99	17.9
Ashkenazi Jewish	10,370	846	0.08	0.16	0.67	15	0.63	14.6
East Asian	19,954	546	0.03	0.05	0.07	5.3	0.05	4.3
Finnish	25,124	1238	0.05	0.10	0.24	9.4	0.24	9.3
European (not Finnish)	129,206	7573	0.06	0.12	0.34	11	0.32	10.6
South Asian	30,616	2840	0.09	0.19	0.86	16.8	0.79	16.2
Other ethnicities	7228	611	0.08	0.17	0.71	15.5	0.67	15
African/African American	24,974	14,458	0.58	1.16	33.52	51	32.49	51
African and African American^b^	24,974	2366	0.09	0.19	0.90	17.2	0.74	15.7

^a^The global prevalence of carriership and recessive *VWF* variants is calculated after excluding the 3 common genetic variant in the African/American ethnicity (p.Arg2185Gln, p.Met740Ile and p.His817Gln).

^b^After excluding p.Arg2185Gln, p.Met740Ile and p.His817Gln variants.

**Table 5:** Estimated global prevalence of autosomal dominant- and recessive von Willebrand disease (VWD).

Population	Type 1 VWD prevalence in 1000 individuals (autosomal dominant), n variant (*n* = 78)	2A VWD prevalence in 1000 individuals (autosomal dominant), n variant (*n* = 23)	2B VWD prevalence in 1000 individuals (autosomal dominant), n variant (*n* = 12)	2M VWD prevalence in 1000 individuals (autosomal dominant), n variant (*n* = 21)	2N VWD^a^ prevalence in 1000 individuals (autosomal recessive), n variant (*n* = 144)	Type 3 VWD^b^ prevalence in 1000 individuals (autosomal recessive), n variant (*n* = 129)
All^c^	74	3	3	6	0.31	0.7
African/African American^d^	65	1	1	9	0.09	2.2
Latino/Admixed American	112	4	4	2.6	0.89	2.1
Ashkenazi Jewish	29	0.4	11	42	0.01	0.1
East Asian	21	10	0.2	1	0.0011	0.1
Finnish	61	1.5	7	10	0.34	0.7
European (not Finnish)	71	2	2	3	0.24	0.3
South Asian	48	1	4	5	0.09	0.7
Other Ethnicities	78	4	4	7	0.43	0.5

^a^The global prevalence of VWD type 2N was calculated using type 1 or 3 variants with type 2N variants, after removing all variants with a MAF > 1%.

^b^The global prevalence of VWD type 3 was calculated using both type 1 and type 3 (*n* = 54) variants, after removing all variants with a MAF > 1%.

^c^The global prevalence of VWD is calculated after excluding the common genetic variants in the African/American ethnicity (p.His817Gln as a type 2N and p.Arg2185Gln as a type 1).

^d^After excluding p.His817Gln and Arg2185Gln variants.

## Discussion

The prevalence of genetic diseases has traditionally been established by observing the disease itself. A number of investigators, who attempted to estimate the prevalence of VWD by counting VWD cases in countries such as Italy, U.S.A., or Canada^16–19^, obtained an estimated prevalence ranging from 0.6 to 1.3%, with 1 in 1000 cases having clinical manifestations. It is noteworthy that all of these studies were limited by relatively small numbers of studied cases and geographic specificity without an accompanying genetic study. A new possibility arose to estimate the global prevalence of a disease with the advent of large databases of population genetic sequencing such as gnomAD^20–22^. The present comprehensive investigation provides a novel and a truly global estimation of VWD prevalence because for the first time, we attempted to estimate global VWD prevalence by using the available genome and exome data from more than 141,000 individuals. We found a prevalence of 13.9% for the gnomAD alleles carrying *VWF* pathogenic variants in the heterozygous state and 0.48% in the recessive form. When considering only reported *VWF* variants, a similar estimation of prevalence was found (13.7% and 0.47%). To calculate the global prevalence of dominant and recessive VWD types, we used the already reported variants associated with VWD as identified in the frame of the gnomAD population (*n* = 218) with a clear autosomal dominant or recessive inheritance. Accordingly, the global prevalence of VWD in 1000 individuals was estimated to be 74 for type 1, 3 for 2A, 3 for 2B and 6 for 2M. The global prevalences for recessive VWD forms (type 2N and type 3) were 0.31 and 0.7 in 1000 individuals, respectively. In addition, it appears that VWD prevalence differs among various populations (Table 5).

The high VWD prevalence established in this large-scale genetic database indicates that the genetic predisposition to develop VWD due to *VWF* variants is likely to be more common than hitherto reported and also highlights that many patients carrying these variants are still not diagnosed. These data provide a hint that VWD is likely to be grossly underdiagnosed worldwide, which could contribute to undertreatment, significant (avoidable) morbidity, and health care system burden. Available data suggests that despite the fact that VWD is common, it is paradoxically underdiagnosed owing to several factors, including complex diagnosis, inaccurate distinction between normal or abnormal bleeding symptoms, relatively mild clinical severity as well as lack of disease awareness among non-specialist healthcare providers^23,24^. We identified 287 novel and potentially pathogenic and 218 previously reported *VWF* variants in the gnomAD population, in which among a total of 282,912 alleles 31,785 carried *VWF* pathogenic variants. In comparison with other ethnicities, the East Asian population was more largely affected by novel variants perhaps because it was previously less investigated, with 18.9% of affected alleles being carriers of the novel variants, whereas only less than 5% was observed in other ethnicities.

VWD results from heterozygous, homozygous or compound heterozygous variants in the *VWF*. We found that of 141,456 individuals in the gnomAD population 1026 (0.72%) were homozygotes for different *VWF* variants, with 29,733 (21%) apparently heterozygotes and 110,697 (78.3%) wild type. Of note, we were unable to determine whether some variants are in compound heterozygosity, because no information is available in this regard in gnomAD. Among the homozygous cases, type 3 VWD variants were found in 6 individuals of African/African American or Latino/Admixed American ethnicities. Homozygosity for type 2N variants was found in 152 Africans/African Americans and in 2 Finnish- and 3 non-Finnish Europeans. The remaining 848 homozygous variants were responsible for type 1, 2A, 2B and 2M or remained unclassified.

There was a remarkably higher number of gnomAD variants (both reported and novel) in D1-D2, A3, D4 and C1-C6 VWF domains than in the HGMD/LOVD datasets. In contrast, the most functional VWF domains exhibited almost similar (D’-D3) or significantly fewer novel variants (A1, A2 and CK). A possible explanation is that the D’, D3, A1, A2, and CK domains are very critical for normal VWF function and hence most of the possible variants in these regions have been already identified. It might also be the result of the target sequencing approach being used until recently for VWD type 2 with defective D’-D3 and A1-A2 domains. In a separate analysis (data not shown), we found that the VWF A1-A2 were the most susceptible domains to nucleotide changes and that only about 20 and 40% of their amino acids are being conserved, i.e., not involved in pathogenic variants.

We depicted a full picture of the VWF domain distribution in different VWD types using all the so-far SNVs or short insertions/deletions reported pathogenic *VWF* variants (*n* = 927). Our data showed that missense variants are responsible for the majority of reported and gnomAD variants, in agreement with established knowledge that the majority of type 1, almost all type 2 and some type 3 VWD are due to missense mutations^12–15^.

We identified at least 5 most frequent ethnic-specific variants in the gnomAD population with an already reported association with VWD. Interestingly, population with African/African American (p.Arg2185Gln, p.Met740Ile, p.His817Gln), Latino/Admixed American (p.Met740Ile, p.Arg2185Gln, p.Pro2063Ser, p.His817Gln) and Ashkenazi Jewish (p.Pro2063Ser, p.Arg2185Gln, p.Met740Ile) ethnicities shared almost the same most frequent variants in gnomAD. However, some recurrent variants were specific of a given population such as p.Gly967Asp and p.Thr1034del in Africans/African Americans, p.Met576Ile in Latinos/Admixed Americans, p.Ser1731Thr and p.Arg2342His in Ashkenazi Jewish. The South and East Asian populations presented a quite different pattern for the most recurrent variants. p.Pro2063Ser, p.Asn1231Ser, c.1730-5 C > T, p.Arg2185Gln and p.Arg924Gln where recurrently observed in South Asians, whereas p.Gly2035Asp, p.Arg2287Gln, p.Gly624Ser, c.2967+2 T > C and p.Gly1672 Arg were frequent in East Asians. The most prevalent variants also were different between Finnish- and non-Finnish European populations except for p.Arg854Gln, p.Arg924Gln and p.Thr2647Met being common in both ethnicities. In the Finnish population, p.Val1439Met and p.Pro1266Leu were common as opposed to non-Finnish where p.Pro2063Ser and p.Tyr1584Cys were recurrent. Given that several of these variants have a MAF > 1 % (Table 6), it is possible that they lead only to a slight reduction of VWF levels or that their phenotypic expression requires the presence of environmental triggers, clinical challenges or additional variants which cosegregate to manifest bleeding^25,26^. This complex scenario may pose clinical challenges in establishing VWD diagnosis in heterozygous carriers of such variants.

**Table 6:** *VWF* variants identified with a minor allele frequency of >1% in at least one ethnicity.

cDNA	Protein Consequence	Type of variant	All	African/African American	Latino/Admixed American	Ashkenazi Jewish	East Asian	Finnish	European	South Asian	Other
c.6554 G > A	p.Arg2185Gln	missense	0.0196	0.1899	0.0106	0.0050	0.0002	0.0001	0.0013	0.0043	0.0089
c.2220 G > A	p.Met740Ile	missense	0.0180	0.1801	0.0117	0.0043	0.0000	0.0000	0.0007	0.0002	0.0076
c.6187 C > T	p.Pro2063Ser	missense	0.0117	0.0012	0.0102	0.0250	0.0001	0.0012	0.0081	0.0485	0.0147
c.2451 T > A	p.His817Gln	missense	0.0112	0.1157	0.0062	0.0000	0.0000	0.0000	0.0002	0.0002	0.0046
c.2771 G > A	p.Arg924Gln	missense	0.0107	0.0032	0.0045	0.0030	0.0001	0.0056	0.0187	0.0037	0.0115
c.1728G>T	p.Met576Ile	missense	0.0050	0.0002	0.0375	0.0000	0.0002	0.0000	0.0002	0.0002	0.0041
c.7940 C > T	p.Thr2647Met	missense	0.0038	0.0007	0.0004	0.0019	0.0002	0.0198	0.0037	0.0003	0.0040
c.2900 G > A	p.Gly967Asp	missense	0.0025	0.0257	0.0016	0.0000	0.0000	0.0000	0.0001	0.0001	0.0008
c.3101_3103del	p.Thr1034del	inframe_deletion	0.0015	0.0152	0.0008	0.0000	0.0000	0.0000	0.0002	0.0001	0.0004
c.5191 T > A	p.Ser1731Thr	missense	0.0015	0.0001	0.0010	0.0209	0.0000	0.0000	0.0011	0.0002	0.0022

In previous genetic studies conducted on African/Americans and white healthy controls^26,27^, p.Met576Ile, p.His817Gln and p.Arg2185Gln were found in more than 15% of African-American controls, while p.Arg854Gln and p.Pro2063Ser were only found in whites. We conducted this global analysis of *VWF* variants to provide background information for understanding the presence of *VWF* variants in disease by using HGMD and LOVD and non-disease populations using gnomAD. Collectively, ours as well as available data^26,27^ highlight that several *VWF* variants are more prevalent than reported, either ethnically or globally. Further studies are therefore necessary in order to determine whether these variants are actually associated with VWD, their penetrance and modifiers of effect, or whether they should be instead classified as benign variants in the corresponding populations. Based on the NHLBI database, reduced VWF and FVIII levels have been already established for p.Arg2185Gln and p.His817Gln, respectively^25^. Available data also suggests that p.Pro2063Ser is a common neutral *VWF* polymorphic variant^28^.

This study has limitations. In silico algorithms have been used to predict the pathogenicity of missense and splicing *VWF* variants. However, to minimize false positives, a restricted approach was implemented using as many as 7 different prediction tools for missense and 4 for splicing variants. Another limitation is that we may have underestimated the number of pathogenic variants, because promoter, deep intronic, insertion and deletion variants are not always recognized by variant calling programs. In addition, gross deletions and rearrangements may go undetected due to systematic biases in exome sequencing. It is possible that some rare pathogenic variants could be missed in exon 26 of *VWF* due to its lower coverage compared to other exons. Finally, no VWF plasma measurements were available to confirm variant pathogenicity. Thus the present data should be interpreted with caution because some of the identified *VWF* variants may not be pathogenic and those already reported may not be fully penetrant. This notwithstanding, we believe that false positives have been minimized since the estimations were similar when both previously reported and novel variants were taken into account and also because we used a very strict classification approach to identify pathogenic variants. While other investigators used a small number of in silico tools^21,29–31^, we used 7 prediction tools for missense and 4 for splicing variants. This strict strategy probably led to the exclusion of several pathogenic variants and therefore the prevalence of VWD could be even higher. Indeed, among the previously reported variants found in the gnomAD, only 30% passed all 7 (for missense) or 3 (for splicing) prediction algorithms.

In conclusion, we have attempted for the first time to estimate the worldwide and within-population prevalence of VWD using available genome and exome sequencing data of 141,456 individuals from the gnomAD. Our study reveals that there is a considerably higher than expected prevalence of putative disease-causing alleles and *VWF* variants associated with VWD. This finding suggests that a large number of VWD patients are perhaps still undiagnosed and thus are undertreatment. Our analysis also provides a global mutational landscape of VWD for old and novel variants.

## Methods

We extracted all identified variants in the *VWF* from the gnomAD (v2.1) which includes 125,748 whole exomes and 15,708 whole genomes from unrelated individuals^32^. These sequence data are part of various disease-specific and population genetic studies, totaling 141,456 individuals and are aligned against the GRCh37/hg19 human genome reference. A wide range of ethnicities is represented in this population-based database. Individuals known to be affected by the severe disease at pediatric age and their first-degree relatives have been removed from this dataset^32^.

Due to the NGS technical limitations for the detection of large insertions, duplication or deletions and complex rearrangements, our analysis focused only on SNV and short insertions/deletions. Among *VWF* genetic variants identified in the gnomAD population, we considered the followings as deleterious:

1. All variants reported to be clearly associated with VWD in the HGMD professional version (our release dates back to 2022) and/or LOVD (accessed 2022) version 2 (https://grenada.lumc.nl/LOVD2/VWF/home.php) and version 3 (https://databases.lovd.nl/shared/variants/VWF);
2. Nonsense, frameshift and inframe deletion or insertion variants;
3. Disruptive splice-site variants affecting the first 2 or last 2 intronic nucleotides;
4. Splice-site variants located at the first 8 or last 8 intronic positions and predicted to be deleterious by 4 of 4 different in silico tools: Varseak (https://varseak.bio/), ESEFinder (http://rulai.cshl.edu/cgi-bin/tools/ESE3/esefinder.cgi?process=home)^33^, BDGP (https://www.fruitfly.org/seq_tools/splice.html)^34^ and CADD (https://cadd.gs.washington.edu/)^35^. The gene variants that abolish the wild-type splicing site or reduce the prediction score to less than half of the wild-type counterpart were considered deleterious and a CADD score of ≥ 20 was considered deleterious;
5. Missense variants that were predicted as deleterious by 7 out of 7 different in silico programs: CADD (https://cadd.gs.washington.edu/)^35^, SIFT (https://sift.bii.a-star.edu.sg/)^36^, PolyPhen2 (http://genetics.bwh.harvard.edu/pph2/)^37^, LRT (https://evomics.org/resources/likelihood-ratio-test/)^38^, MutationTaster (https://www.mutationtaster.org/)^39^, MutationAssessor (http://mutationassessor.org/r3/)^40^ and FATHMM (https://fathmm.biocompute.org.uk/fathmmMKL.htm)^41^.

To portray the global mutational landscape of VWD, we mined and analyzed all *VWF* variants associated with VWD in the HGMD and LOVD genetic database. The comparison of the results obtained from the gnomAD with those stemming from the two forementioned disease genetic databases was further performed. In order to classify variants related to VWD according to a specific phenotype, we referred to the HGMD and LOVD classifications, but in case of discrepancy, the published paper concerning the given gene variant was considered. UC variants are those found in patients for whom no clear VWD type has been established or when controversial classification has been reported in the literature. Variants reported in the HGMD and LOVD datasets without a clear association with VWD have been removed from the analysis.

To calculate the worldwide prevalence of VWD, we applied two different approaches. First, we considered all gnomAD variants identified as pathogenic according to the aforementioned approach. The second approach was to limit the analysis only to those variants identified in the gnomAD that have been previously described to be clearly associated with VWD in the available genetic databases (i.e., HGMD and LOVD). We calculated the estimated prevalence of VWD using the Hardy-Weinberg equation (p^2^ + 2pq + q^2^ = 1), where p is the population frequency of the major allele and q is the population frequency of the minor allele.

The frequencies of all possible haplotypes generated by common variants identified in different populations of 1000 Genomes project was evaluated using LDhap application (LDlink suite - https://ldlink.nci.nih.gov/?tab=home).

### Reporting summary

Further information on research design is available in the Nature Research Reporting Summary linked to this article.

### Supplementary information

Supplementary Materials

Reporting summary

## Data Availability

The exome and genome sequencing data of the VWF gene (VCF) can be downloaded from gnomAD for free. All identified variants in gnomAD that were classified as pathogenic are available in the Supplementary Tables 1 and 2. The final datasets analyzed during the current study are available from the corresponding author (flora.peyvandi@unimi.it) upon reasonable request.

## References

[1] Ruggeri ZM (1997). von Willebrand factor. J. Clin. Investig..

[2] Mojzisch A, Brehm MA (2021). The manifold cellular functions of von willebrand factor. Cells.

[3] Zhou YF (2012). Sequence and structure relationships within von Willebrand factor. Blood.

[4] Peyvandi F, Garagiola I, Baronciani L (2011). Role of von Willebrand factor in the haemostasis. Blood Transfus..

[5] Lenting PJ, Casari C, Christophe OD, Denis CV (2012). von Willebrand factor: the old, the new and the unknown. J. Thrombosis Haemost..

[6] Ginsburg D (1985). Human von Willebrand factor (vWF): isolation of complementary DNA (cDNA) clones and chromosomal localization. Science.

[7] Lynch DC (1985). Livingston, Molecular cloning of cDNA for human von Willebrand factor: authentication by a new method. Cell.

[8] Sadler JE (1985). Cloning and characterization of two cDNAs coding for human von Willebrand factor. Proc. Natl. Acad. Sci. USA.

[9] Verweij CL (1985). Construction of cDNA coding for human von Willebrand factor using antibody probes for colony screening and mapping of the chromosomal gene. Nucleic Acids Res..

[10] Mancuso DJ (1991). Human von Willebrand factor gene and pseudogene: structural analysis and differentiation by polymerase chain reaction. Biochemistry.

[11] Sadler JE (2006). Update on the pathophysiology and classification of von Willebrand disease: a report of the Subcommittee on von Willebrand Factor. JTH.

[12] de Jong A, Eikenboom J (2017). Von Willebrand disease mutation spectrum and associated mutation mechanisms. Thrombosis Res..

[13] Baronciani L (2021). Genotypes of European and Iranian patients with type 3 von Willebrand disease enrolled in 3WINTERS-IPS. Blood Adv..

[14] Christopherson PA (2022). Molecular pathogenesis and heterogeneity in type 3 VWD families in U.S. Zimmerman program. J. Thromb. Haemost..

[15] Seidizadeh O (2022). Phenotypic and genetic characterization of the Milan cohort of von Willebrand disease type 2. Blood Adv..

[16] Rodeghiero F, Castaman G, Dini E (1987). Epidemiological investigation of the prevalence of von Willebrand’s disease. Blood.

[17] Werner EJ (1993). Prevalence of von Willebrand disease in children: a multiethnic study. J. Pediatr..

[18] Bowman M, Hopman WM, Rapson D, Lillicrap D, James P (2010). The prevalence of symptomatic von Willebrand disease in primary care practice. J. Thrombosis Haemost..

[19] Bloom, Al. von Willebrand factor: clinical features of inherited and acquired disorders. In *Mayo Clinic Proceedings* 1991 Jul 1 (Vol. 66, pp. 743–751). 10.1016/s0025-6196(12)62088-6 2072762

[20] Pugh J (2019). Use of big data to estimate prevalence of defective DNA repair variants in the US Population. JAMA Dermatol..

[21] Hughes BG, Harrison PM, Hekimi S (2017). Estimating the occurrence of primary ubiquinone deficiency by analysis of large-scale sequencing data. Sci. Rep..

[22] Asselta R (2017). Exploring the global landscape of genetic variation in coagulation factor XI deficiency. Blood.

[23] Sidonio RF, Haley KM, Fallaize D (2017). Impact of diagnosis of von Willebrand disease on patient outcomes: Analysis of medical insurance claims data. Haemophilia.

[24] Corrales-Medina FF (2023). A need to increase von Willebrand disease awareness: vwdtest.com - A global initiative to help address this gap. Blood Rev..

[25] Johnsen JM (2013). NHLBI Exome Sequencing Project. Common and rare von Willebrand factor (VWF) coding variants, VWF levels, and factor VIII levels in African Americans: the NHLBI Exome Sequencing Project. Blood.

[26] Wang QY (2013). Characterizing polymorphisms and allelic diversity of von W illebrand factor gene in the 1000 Genomes. J. Thrombosis Haemost..

[27] Bellissimo DB (2012). VWF mutations and new sequence variations identified in healthy controls are more frequent in the African-American population. Blood.

[28] Hampshire DJ (2014). Goodeve AC. p.P2063S: a neutral VWF variant masquerading as a mutation. Ann. Hematol..

[29] Jian X, Boerwinkle E, Liu X (2014). In silico tools for splicing defect prediction: a survey from the viewpoint of end users. Genet. Med..

[30] Kaler SG, Ferreira CR, Yam LS (2020). Estimated birth prevalence of Menkes disease and ATP7A-related disorders based on the Genome Aggregation Database (gnomAD). Mol. Genet. Metab. Rep..

[31] Soussi T, Leroy B, Devir M, Rosenberg S (2019). High prevalence of cancer‐associated TP53 variants in the gnomAD database: A word of caution concerning the use of variant filtering. Hum. Mutat..

[32] Karczewski KJ (2020). The mutational constraint spectrum quantified from variation in 141,456 humans. Nature.

[33] Cartegni L, Wang J, Zhu Z, Zhang MQ, Krainer AR (2003). ESEfinder: a web resource to identify exonic splicing enhancers. Nucleic Acid Res..

[34] Reese MG, Eeckman FH, Kulp D, Haussler D (1997). Improved splice site detection in genie. J. Comp. Biol..

[35] Rentzsch P, Schubach M, Shendure J, Kircher M (2021). CADD-Splice—improving genome-wide variant effect prediction using deep learning-derived splice scores. Genome Med..

[36] Kumar P, Henikoff S, Ng PC (2009). Predicting the effects of coding non-synonymous variants on protein function using the SIFT algorithm. Nat. Protoc..

[37] Adzhubei IA (2010). A method and server for predicting damaging missense mutations. Nat. Methods.

[38] Felsenstein J (1981). Evolutionary trees from DNA sequences: a maximum likelihood approach. J. Mol. Evol..

[39] Schwarz JM, Rodelsperger C, Schuelke M, Seelow D (2010). MutationTaster evaluates disease-causing potential of sequence alterations. Nat. Methods.

[40] Reva B, Antipin Y, Sander C (2011). Predicting the functional impact of protein mutations: Application to cancer genomics. Nucleic Acids Res..

[41] Shihab HA (2013). Predicting the functional, molecular, and phenotypic consequences of amino acid substitutions using hidden Markov models. Hum. Mutat..

