# Supplementary material for: Population-based prevalence and mutational landscape of von Willebrand disease using large-scale genetic databases
Source: NPJ Genom Med. 2023 Oct 16;8:31. doi: 10.1038/s41525-023-00375-8 (PMC10579253; doi:10.1038/s41525-023-00375-8)
Supplement: Supplementary file 1 — Supplementary Materials [file 41525_2023_375_MOESM1_ESM.pdf]

Supplementary Materials

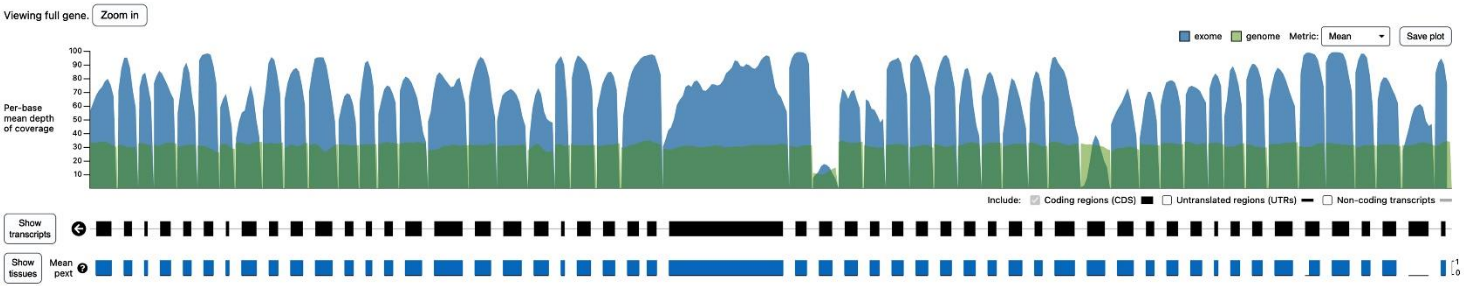

Supplementary Figure 1. Mean depth of coverage per base of *VWF* for exome (blue) and genome (green) data of the gnomAD database (https://gnomad.broadinstitute.org/gene/ENSG00000110799?dataset=gnomad_r2_1).

RS Number	Position (GRCh37)	Allele Frequencies	Haplotypes							
rs2229446	chr12:6103072	C=0.757, T=0.243	C	T	T	C	C	T	T	C
rs57950734	chr12:6145649	A=0.844, T=0.156	A	A	T	A	T	A	T	T
rs2228317	chr12:6155950	C=0.781, T=0.219	C	C	T	T	T	T	C	C
		Haplotype Count	843	146	106	87	56	40	29	15
		Haplotype Frequency	0.6377	0.1104	0.0802	0.0658	0.0424	0.0303	0.0219	0.0113

Supplementary Figure 2. Linkage disequilibrium analysis for the 3 common variants identified in the African/American ethnicity including p.Arg2185Gln (rs2229446), p.Met740Ile (rs2228317) and p.His817Gln (rs57950734).

Supplementary Table 1. Novel variants found in the gnomAD predicted to be pathogenic.

Transcript Consequence	Protein Consequence	rsIDs	Type of variant	Allele Frequency	CADD	SIFT	Polyphen2 HDIV	LRT	Mutation Taster	Mutation Assessor	Fathmm MKI	-2 to 2 nucleotide position[$]	BDGP	ESE finder	Varseak
c.8146T>C	p.Cys2716Arg	rs763365172	missense	3.98E-06	25.9	D	D	D	D	M	D	.	.	.	.
c.7757G>C	p.Gly2586Ala	rs781531963	missense	3.98E-06	23.9	D	P	D	D	M	D	.	.	.	.
c.7720T>C	p.Cys2574Arg	rs1475565311	missense	3.18E-05	24.3	D	D	D	D	M	D	.	.	.	.
c.7712G>T	p.Cys2571Phe	rs1258347142	missense	8.02E-06	25.6	D	D	D	D	M	D	.	.	.	.
c.7678G>A	p.Gly2560Ser	rs749106300	missense	7.08E-06	25.1	D	D	D	D	M	D	.	.	.	.
c.7673C>T	p.Pro2558Leu	rs373158962	missense	1.06E-05	25	D	D	D	D	M	D	.	.	.	.
c.7672C>T	p.Pro2558Ser	rs202068340	missense	7.08E-06	24.4	D	D	D	D	M	D	.	.	.	.
c.7643C>T	p.Ser2548Phe	rs1473847291	missense	3.98E-06	27	D	D	D	D	M	D	.	.	.	.
c.7579C>T	p.Pro2527Ser	rs758376829	missense	3.58E-05	25.2	D	D	D	D	M	D	.	.	.	.
c.7579C>A	p.Pro2527Thr	rs758376829	missense	3.98E-06	24.7	D	D	D	D	M	D	.	.	.	.
c.7525G>T	p.Asp2509Tyr	rs757915054	missense	3.98E-06	24.6	D	D	D	D	M	D	.	.	.	.
c.7525G>A	p.Asp2509Asn	rs757915054	missense	8.85E-05	24.4	D	D	D	D	M	D	.	.	.	.
c.7496G>A	p.Cys2499Tyr	rs1416647748	missense	3.98E-06	27.6	D	D	D	D	M	D	.	.	.	.
c.7468T>C	p.Cys2490Arg	rs747351776	missense	3.98E-06	28.2	D	D	D	D	M	D	.	.	.	.
c.7465G>A	p.Glu2489Lys	rs769241795	missense	2.79E-05	31	D	D	D	D	M	D	.	.	.	.
c.7284C>A	p.Asp2428Glu	rs764400779	missense	3.98E-06	20	D	P	D	D	M	D	.	.	.	.
c.7282G>A	p.Asp2428Asn	rs754363583	missense	1.19E-05	25.9	D	D	D	D	M	D	.	.	.	.
c.7270A>G	p.Thr2424Ala	rs765740267	missense	3.98E-06	22	D	P	D	D	M	D	.	.	.	.
c.7268C>A	p.Thr2423Asn	rs957015244	missense	3.98E-06	25	D	D	D	D	M	D	.	.	.	.
c.7258T>A	p.Cys2420Ser	rs758495824	missense	3.98E-06	27.2	D	D	D	D	M	D	.	.	.	.
c.7253G>A	p.Cys2418Tyr	rs780157825	missense	7.95E-06	29	D	D	D	D	M	D	.	.	.	.
c.7247A>G	p.Asn2416Ser	rs747165661	missense	1.59E-05	25.6	D	D	D	D	M	D	.	.	.	.
c.7244C>T	p.Thr2415Ile	rs1374179815	missense	3.19E-05	25.6	D	D	D	D	M	D	.	.	.	.
c.7212C>G	p.Ser2404Arg	rs759137155	missense	3.98E-06	26.4	D	P	D	D	M	D	.	.	.	.
c.7211G>A	p.Ser2404Asn	rs767130840	missense	3.98E-06	21.8	D	P	D	D	M	D	.	.	.	.
c.7210A>C	p.Ser2404Arg	rs777016887	missense	1.59E-05	26.5	D	P	D	D	M	D	.	.	.	.
c.7199A>C	p.Asn2400Thr	rs750888785	missense	1.19E-05	25	D	P	D	D	M	D	.	.	.	.

c.7181G>A	p.Cys2394Tyr	rs1288690774	missense	3.19E-05	29.3	D	D	D	D	M	D	.	.	.	.
c.7179G>C	p.Glu2393Asp	rs1403529326	missense	3.98E-06	23.7	D	P	D	D	M	D	.	.	.	.
c.7178A>C	p.Glu2393Ala	rs766444913	missense	7.96E-06	27.5	D	P	D	D	M	D	.	.	.	.
c.7060T>G	p.Cys2354Gly	rs1439719475	missense	4.00E-06	28.5	D	D	D	D	M	D	.	.	.	.
c.7051C>A	p.Pro2351Thr	rs1339970856	missense	3.99E-06	25.8	D	D	D	D	M	D	.	.	.	.
c.7028G>A	p.Gly2343Asp	rs61750629	missense	3.99E-06	25.5	D	D	D	D	M	D	.	.	.	.
c.6956G>A	p.Cys2319Tyr	rs1417651109	missense	3.98E-06	26	D	D	D	D	M	D	.	.	.	.
c.6920G>A	p.Cys2307Tyr	rs1317722592	missense	3.98E-06	25	D	D	D	D	M	D	.	.	.	.
c.6919T>C	p.Cys2307Arg	rs1241346987	missense	3.98E-06	24.8	D	D	D	D	M	D	.	.	.	.
c.6893C>T	p.Thr2298Met	rs774051063	missense	0.000106417	27.9	D	D	D	D	M	D	.	.	.	.
c.6875C>A	p.Thr2292Lys	rs755358165	missense	3.98E-06	25.2	D	D	D	D	M	D	.	.	.	.
c.6874A>G	p.Thr2292Ala	rs540687510	missense	3.98E-06	24.3	D	D	D	D	M	D	.	.	.	.
c.6872G>C	p.Cys2291Ser	rs753138371	missense	3.98E-06	24.9	D	D	D	D	M	D	.	.	.	.
c.6829C>G	p.Pro2277Ala	rs767257065	missense	3.98E-06	25.7	D	D	D	D	M	D	.	.	.	.
c.6784G>A	p.Gly2262Arg	rs147715696	missense	3.98E-06	25.9	D	D	D	D	M	D	.	.	.	.
c.6768G>C	p.Gln2256His	rs200300292	missense	2.83E-05	24.4	D	D	D	D	M	D	.	.	.	.
c.6680G>T	p.Cys2227Phe	rs777023647	missense	1.59E-05	31	D	D	D	D	H	D	.	.	.	.
c.6611C>T	p.Pro2204Leu	rs777556669	missense	3.98E-05	25.1	D	D	D	D	H	D	.	.	.	.
c.6562G>A	p.Gly2188Arg	rs1036690966	missense	7.99E-06	25.8	D	D	D	D	H	D	.	.	.	.
c.6541G>A	p.Ala2181Thr	rs771544504	missense	1.20E-05	29.3	D	D	D	D	M	D	.	.	.	.
c.6475T>C	p.Phe2159Leu	rs534005857	missense	3.98E-06	28.5	D	D	D	D	H	D	.	.	.	.
c.6452A>G	p.His2151Arg	rs1330984328	missense	3.98E-06	25.7	D	D	D	D	M	D	.	.	.	.
c.6358G>A	p.Gly2120Arg	rs1437238974	missense	3.98E-06	24.4	D	D	D	D	M	D	.	.	.	.
c.6287T>A	p.Phe2096Tyr	rs762847913	missense	3.19E-05	27	D	D	D	D	M	D	.	.	.	.
c.6284A>G	p.Asp2095Gly	rs1406083255	missense	3.99E-06	26.2	D	D	D	D	M	D	.	.	.	.
c.6281A>G	p.Asn2094Ser	rs199546602	missense	1.60E-05	24.9	D	D	D	D	M	D	.	.	.	.
c.6265G>T	p.Asp2089Tyr	rs984855564	missense	2.49E-05	28.7	D	D	D	D	M	D	.	.	.	.
c.6265G>C	p.Asp2089His	rs984855564	missense	4.00E-06	27.6	D	D	D	D	M	D	.	.	.	.
c.6247G>A	p.Gly2083Ser	rs752022525	missense	3.98E-06	25.3	D	D	D	D	M	D	.	.	.	.
c.6170A>C	p.His2057Pro	rs1212564306	missense	3.98E-06	24.5	D	D	D	D	M	D	.	.	.	.

c.6131G>T	p.Gly2044Val	rs763260567	missense	1.59E-05	24.3	D	D	D	D	M	D	.	.	.	.
c.6095C>T	p.Pro2032Leu	rs1354387158	missense	7.96E-06	24.9	D	D	D	D	M	D	.	.	.	.
c.5947G>T	p.Asp1983Tyr	rs748986187	missense	2.39E-05	29.5	D	D	D	D	M	D	.	.	.	.
c.5878A>G	p.Thr1960Ala	rs762012588	missense	7.97E-06	26	D	D	D	D	M	D	.	.	.	.
c.5848T>G	p.Cys1950Gly	rs1306586341	missense	3.18E-05	26.8	D	D	D	D	M	D	.	.	.	.
c.5822G>T	p.Gly1941Val	rs1180283088	missense	4.13E-06	26.3	D	D	D	D	M	D	.	.	.	.
c.5818T>C	p.Cys1940Arg	rs764001779	missense	8.18E-06	27.1	D	D	D	D	M	D	.	.	.	.
c.5816C>A	p.Thr1939Asn	rs561950510	missense	1.22E-05	24.5	D	D	D	D	M	D	.	.	.	.
c.5744A>T	p.His1915Leu	rs532551665	missense	3.99E-06	23.4	D	P	D	D	M	D	.	.	.	.
c.5723G>C	p.Gly1908Ala	rs761406405	missense	8.04E-06	26.3	D	D	D	D	M	D	.	.	.	.
c.5701A>G	p.Thr1901Ala	rs1240823988	missense	4.11E-06	24.6	D	D	D	D	M	D	.	.	.	.
c.5644G>A	p.Glu1882Lys	rs1191180569	missense	3.98E-06	32	D	D	D	D	M	D	.	.	.	.
c.5642A>G	p.Asp1881Gly	rs780276684	missense	3.98E-06	33	D	D	D	D	M	D	.	.	.	.
c.5566G>A	p.Asp1856Asn	NA	missense	3.98E-06	25.1	D	D	D	D	M	D	.	.	.	.
c.5540A>G	p.Asn1847Ser	rs766858874	missense	3.98E-06	22.6	D	D	D	D	M	D	.	.	.	.
c.5483G>T	p.Gly1828Val	rs1299445904	missense	3.98E-06	32	D	D	D	D	M	D	.	.	.	.
c.5482G>A	p.Gly1828Arg	rs763246893	missense	7.96E-06	29.5	D	D	D	D	M	D	.	.	.	.
c.5443G>A	p.Ala1815Thr	rs754062706	missense	1.19E-05	27	D	D	D	D	M	D	.	.	.	.
c.5359G>A	p.Gly1787Ser	rs774768100	missense	3.98E-06	26.5	D	D	D	D	M	D	.	.	.	.
c.5311G>C	p.Gly1771Arg	rs370016586	missense	4.00E-06	36	D	D	D	D	H	D	.	.	.	.
c.5143A>G	p.Lys1715Glu	rs563959776	missense	1.19E-05	26.1	D	D	D	D	M	D	.	.	.	.
c.5139C>G	p.Phe1713Leu	rs150873446	missense	3.98E-06	22.7	D	D	D	D	M	D	.	.	.	.
c.5129T>C	p.Met1710Thr	rs375946218	missense	1.19E-05	25.4	D	D	D	D	M	D	.	.	.	.
c.5099C>T	p.Ser1700Phe	rs369529543	missense	7.96E-06	25	D	D	D	D	M	D	.	.	.	.
c.5092G>T	p.Gly1698Cys	rs760973223	missense	3.18E-05	28.7	D	D	D	D	H	D	.	.	.	.
c.5065C>A	p.Pro1689Thr	rs139320345	missense	2.48E-05	24.5	D	P	D	D	M	D	.	.	.	.
c.5032C>T	p.Pro1678Ser	rs1477870096	missense	3.19E-05	22.7	D	D	D	D	M	D	.	.	.	.
c.5007C>G	p.Cys1669Trp	rs1468180315	missense	4.02E-06	22.8	D	D	D	D	M	D	.	.	.	.
c.4859C>A	p.Pro1620His	rs1254310454	missense	1.59E-05	25.2	D	D	D	D	M	D	.	.	.	.
c.4560C>G	p.Phe1520Leu	rs1257255818	missense	7.97E-06	23.9	D	D	D	D	H	D	.	.	.	.

c.4286A>G	p.Glu1429Gly	rs761350219	missense	3.98E-06	24.3	D	D	D	D	H	D	.	.	.	.
c.4285G>A	p.Glu1429Lys	rs371554756	missense	1.06E-05	24.3	D	D	D	D	M	D	.	.	.	.
c.4282A>G	p.Ile1428Val	rs762661828	missense	1.06E-05	23.5	D	P	D	D	H	D	.	.	.	.
c.4222A>C	p.Lys1408Gln	rs141990425	missense	7.97E-06	25.8	D	D	D	D	H	D	.	.	.	.
c.4130C>T	p.Ala1377Val	rs141211612	missense	0.000255165	23.8	D	D	D	D	H	D	.	.	.	.
c.4051G>A	p.Gly1351Ser	rs1338948803	missense	7.98E-06	28.5	D	D	D	D	H	D	.	.	.	.
c.4025G>C	p.Arg1342Pro	rs527483960	missense	3.99E-06	26.3	D	D	D	D	H	D	.	.	.	.
c.4025G>A	p.Arg1342His	rs527483960	missense	1.20E-05	24	D	P	D	D	M	D	.	.	.	.
c.4003A>G	p.Lys1335Glu	rs1253742420	missense	3.99E-06	24.4	D	D	D	D	H	D	.	.	.	.
c.3931C>A	p.Gln1311Lys	rs267607337	missense	3.98E-06	25.4	D	D	D	D	H	D	.	.	.	.
c.3914A>C	p.Glu1305Ala	rs1265932750	missense	3.98E-06	28.1	D	D	D	D	H	D	.	.	.	.
c.3911T>C	p.Met1304Thr	rs767135898	missense	3.98E-06	26.1	D	D	D	D	H	D	.	.	.	.
c.3868G>A	p.Glu1290Lys	rs138900040	missense	0.000251342	24.9	D	D	D	D	M	D	.	.	.	.
c.3854C>G	p.Ser1285Cys	rs61749380	missense	3.98E-06	27.9	D	D	D	D	H	D	.	.	.	.
c.3838T>G	p.Phe1280Val	rs1370175388	missense	3.19E-05	27.2	D	D	D	D	H	D	.	.	.	.
c.3791A>C	p.Glu1264Ala	rs1367762735	missense	4.00E-06	22.8	D	D	D	D	M	D	.	.	.	.
c.3764C>A	p.Thr1255Asn	rs1042075297	missense	7.14E-06	23.3	D	D	D	D	M	D	.	.	.	.
c.3664T>C	p.Cys1222Arg	rs1166400139	missense	3.98E-06	28.2	D	D	D	D	M	D	.	.	.	.
c.3608G>A	p.Gly1203Asp	rs774790905	missense	3.98E-06	23.5	D	D	D	D	M	D	.	.	.	.
c.3588T>G	p.Cys1196Trp	rs761144403	missense	3.98E-06	23.1	D	D	D	D	H	D	.	.	.	.
c.3539G>T	p.Gly1180Val	rs780855722	missense	1.19E-05	32	D	D	D	D	H	D	.	.	.	.
c.3539G>A	p.Gly1180Glu	rs780855722	missense	3.98E-06	31	D	D	D	D	M	D	.	.	.	.
c.3533C>T	p.Pro1178Leu	rs1388099496	missense	3.71E-05	28.3	D	D	D	D	M	D	.	.	.	.
c.3433C>G	p.Arg1145Gly	rs1206499921	missense	1.84E-05	26.2	D	D	D	D	M	D	.	.	.	.
c.3401A>C	p.Asn1134Thr	rs1239632320	missense	1.85E-05	25.2	D	D	D	D	M	D	.	.	.	.
c.3391G>A	p.Glu1131Lys	rs1029092876	missense	6.45E-05	29.1	D	D	D	D	M	D	.	.	.	.
c.3379C>T	p.Pro1127Ser	rs139579968	missense	0.000135441	27.9	D	D	D	D	M	D	.	.	.	.
c.3335C>T	p.Ala1112Val	rs1408359593	missense	3.18E-05	28.3	D	D	D	D	M	D	.	.	.	.
c.3286G>A	p.Asp1096Asn	rs748673885	missense	2.43E-05	27.9	D	D	D	D	M	D	.	.	.	.
c.3284G>T	p.Gly1095Val	rs1397155952	missense	4.04E-06	26	D	D	D	D	M	D	.	.	.	.

c.3251G>A	p.Cys1084Tyr	rs759805079	missense	8.03E-06	24.6	D	D	D	D	H	D	.	.	.	.
c.3236C>T	p.Pro1079Leu	rs1334197148	missense	4.03E-06	22.8	D	P	D	D	M	D	.	.	.	.
c.3092G>A	p.Cys1031Tyr	rs141412860	missense	7.96E-06	29.2	D	D	D	D	H	D	.	.	.	.
c.3067A>G	p.Asn1023Asp	rs1395402253	missense	3.98E-06	26.7	D	D	D	D	M	D	.	.	.	.
c.3029G>A	p.Ser1010Asn	rs751539842	missense	1.19E-05	27.4	D	D	D	D	M	D	.	.	.	.
c.3014A>G	p.Asn1005Ser	rs749285654	missense	3.98E-06	25.6	D	D	D	D	M	D	.	.	.	.
c.3002G>C	p.Gly1001Ala	rs1476253383	missense	7.96E-06	26.7	D	D	D	D	M	D	.	.	.	.
c.2993A>G	p.Asn998Ser	rs1369988754	missense	3.18E-05	25.5	D	D	D	D	H	D	.	.	.	.
c.2992A>C	p.Asn998His	rs1438192210	missense	3.19E-05	26.7	D	D	D	D	H	D	.	.	.	.
c.2588G>T	p.Cys863Phe	rs1344449213	missense	3.98E-06	28	D	D	D	D	H	D	.	.	.	.
c.2540A>G	p.Asn847Ser	rs748704250	missense	7.95E-06	23.7	D	D	D	D	M	D	.	.	.	.
c.2536T>G	p.Cys846Gly	rs200106723	missense	0.000109595	24.3	D	D	D	D	H	D	.	.	.	.
c.2515G>A	p.Gly839Arg	rs1373658393	missense	3.18E-05	27.4	D	D	D	D	H	D	.	.	.	.
c.2492A>G	p.His831Arg	rs1250869827	missense	7.95E-06	25.1	D	D	D	D	M	D	.	.	.	.
c.2482C>T	p.Pro828Ser	rs1285074430	missense	0.000127372	24.5	D	D	D	D	M	D	.	.	.	.
c.2450A>G	p.His817Arg	rs752443680	missense	1.19E-05	24.7	D	P	D	D	M	D	.	.	.	.
c.2312T>C	p.Met771Thr	rs199588249	missense	1.19E-05	24.7	D	P	D	D	M	D	.	.	.	.
c.2173C>T	p.His725Tyr	rs770830710	missense	3.98E-06	28.5	D	D	D	D	M	D	.	.	.	.
c.2170G>C	p.Asp724His	rs1448001886	missense	3.98E-06	28.9	D	D	D	D	M	D	.	.	.	.
c.2146T>A	p.Phe716Ile	rs771858185	missense	3.98E-06	27.3	D	D	D	D	M	D	.	.	.	.
c.2138G>A	p.Gly713Asp	rs760218536	missense	2.39E-05	26.6	D	D	D	D	M	D	.	.	.	.
c.2126G>A	p.Cys709Tyr	rs1216992284	missense	3.98E-06	27	D	D	D	D	M	D	.	.	.	.
c.2108C>G	p.Pro703Arg	rs377240952	missense	3.98E-06	24.8	D	D	D	D	M	D	.	.	.	.
c.2074G>C	p.Gly692Arg	rs1398231658	missense	3.98E-06	27.9	D	D	D	D	H	D	.	.	.	.
c.2048G>A	p.Cys683Tyr	rs764286793	missense	3.98E-06	27	D	D	D	D	H	D	.	.	.	.
c.2036G>A	p.Cys679Tyr	rs1457741631	missense	3.98E-06	27.5	D	D	D	D	H	D	.	.	.	.
c.1994G>A	p.Cys665Tyr	rs1387523104	missense	3.98E-06	28.4	D	D	D	D	H	D	.	.	.	.
c.1984G>A	p.Gly662Arg	rs772573308	missense	2.13E-05	29.1	D	D	D	D	M	D	.	.	.	.
c.1981T>G	p.Cys661Gly	rs1401763967	missense	7.97E-06	27.5	D	D	D	D	H	D	.	.	.	.
c.1958C>T	p.Pro653Leu	rs11064012	missense	3.22E-05	26	D	D	D	D	M	D	.	.	.	.

c.1862G>T	p.Cys621Phe	rs886049744	missense	7.55E-06	29.6	D	D	D	D	M	D	.	.	.	.
c.1831G>T	p.Asp611Tyr	rs1327495128	missense	3.19E-05	32	D	D	D	D	H	D	.	.	.	.
c.1618G>A	p.Gly540Arg	rs1364511255	missense	7.98E-06	27.7	D	D	D	D	M	D	.	.	.	.
c.1552G>A	p.Gly518Arg	rs767005834	missense	4.02E-06	28.2	D	D	D	D	M	D	.	.	.	.
c.1517G>A	p.Gly506Glu	rs771947987	missense	8.03E-06	29	D	D	D	D	M	D	.	.	.	.
c.1516G>C	p.Gly506Arg	rs201456647	missense	3.19E-05	29.7	D	D	D	D	M	D	.	.	.	.
c.1516G>A	p.Gly506Arg	rs201456647	missense	4.01E-05	31	D	D	D	D	M	D	.	.	.	.
c.1510G>T	p.Gly504Cys	rs1249623519	missense	4.01E-06	29.3	D	D	D	D	M	D	.	.	.	.
c.1433G>A	p.Gly478Asp	rs761973219	missense	7.48E-05	25.4	D	D	D	D	M	D	.	.	.	.
c.1432G>C	p.Gly478Arg	rs763102998	missense	3.99E-06	33	D	D	D	D	M	D	.	.	.	.
c.1324C>T	p.Arg442Cys	rs148247755	missense	6.75E-05	26	D	D	D	D	H	D	.	.	.	.
c.1321A>G	p.Thr441Ala	rs1334521989	missense	4.00E-06	23.5	D	D	D	D	M	D	.	.	.	.
c.1312G>A	p.Ala438Thr	rs756620872	missense	1.20E-05	25.1	D	D	D	D	M	D	.	.	.	.
c.1285A>G	p.Thr429Ala	rs1228345297	missense	3.98E-06	24.8	D	D	D	D	M	D	.	.	.	.
c.1220G>T	p.Ser407Ile	rs1350078273	missense	3.98E-06	26.2	D	P	D	D	M	D	.	.	.	.
c.1213A>G	p.Thr405Ala	rs1438233482	missense	3.98E-06	25.3	D	D	D	D	M	D	.	.	.	.
c.1186T>C	p.Phe396Leu	rs780724123	missense	3.98E-06	26.3	D	D	D	D	M	D	.	.	.	.
c.1157G>T	p.Gly386Val	rs367556535	missense	3.98E-06	32	D	D	D	D	M	D	.	.	.	.
c.1151G>A	p.Cys384Tyr	rs775101417	missense	3.98E-06	25.7	D	D	D	D	H	D	.	.	.	.
c.1150T>C	p.Cys384Arg	rs762146804	missense	1.20E-05	23.7	D	D	D	D	H	D	.	.	.	.
c.1130G>T	p.Trp377Leu	rs1321160729	missense	3.98E-06	32	D	D	D	D	H	D	.	.	.	.
c.1078G>T	p.Gly360Cys	rs199570108	missense	3.98E-06	23.4	D	D	D	D	H	D	.	.	.	.
c.1078G>A	p.Gly360Ser	rs199570108	missense	0.000116732	23	D	D	D	D	H	D	.	.	.	.
c.1061G>A	p.Gly354Glu	rs775002681	missense	3.98E-06	25.2	D	D	D	D	M	D	.	.	.	.
c.1060G>A	p.Gly354Arg	rs760061887	missense	1.41E-05	25.9	D	D	D	D	M	D	.	.	.	.
c.1055A>G	p.His352Arg	rs760642864	missense	3.98E-06	23.2	D	D	D	D	M	D	.	.	.	.
c.998A>G	p.Glu333Gly	rs746407466	missense	3.99E-06	25.9	D	P	D	D	M	D	.	.	.	.
c.994C>T	p.Pro332Ser	rs780678562	missense	3.98E-06	25.2	D	P	D	D	M	D	.	.	.	.
c.994C>G	p.Pro332Ala	rs780678562	missense	3.19E-05	23.3	D	D	D	D	M	D	.	.	.	.
c.992G>A	p.Cys331Tyr	rs140004667	missense	7.96E-06	28.6	D	D	D	D	H	D	.	.	.	.

c.973T>C	p.Cys325Arg	rs1352009704	missense	3.98E-06	25.4	D	D	D	D	H	D	.	.	.	.
c.963T>G	p.Cys321Trp	rs762870016	missense	3.19E-05	25.4	D	D	D	D	H	D	.	.	.	.
c.932C>T	p.Thr311Ile	rs201496129	missense	2.78E-05	23.9	D	D	D	D	H	D	.	.	.	.
c.931A>G	p.Thr311Ala	rs777628949	missense	1.59E-05	24.6	D	P	D	D	H	D	.	.	.	.
c.887C>T	p.Pro296Leu	rs1340753881	missense	3.98E-06	25.5	D	D	D	D	M	D	.	.	.	.
c.884G>A	p.Cys295Tyr	rs770781601	missense	3.98E-06	25.1	D	D	D	D	H	D	.	.	.	.
c.872G>T	p.Cys291Phe	NA	missense	4.00E-06	28.9	D	D	D	D	H	D	.	.	.	.
c.871T>G	p.Cys291Gly	rs1466559078	missense	3.19E-05	24.8	D	D	D	D	H	D	.	.	.	.
c.599G>T	p.Cys200Phe	rs1309327221	missense	6.37E-05	23.4	D	D	D	D	H	D	.	.	.	.
c.547G>T	p.Asp183Tyr	rs1189316912	missense	3.98E-06	25.6	D	D	D	D	M	D	.	.	.	.
c.349G>A	p.Gly117Arg	rs748190942	missense	3.18E-05	28.8	D	D	D	D	M	D	.	.	.	.
c.334C>T	p.Pro112Ser	rs1280452644	missense	3.98E-06	25.6	D	D	D	D	M	D	.	.	.	.
c.329C>T	p.Ser110Phe	rs769955003	missense	2.39E-05	25.5	D	D	D	D	M	D	.	.	.	.
c.171C>G	p.Cys57Trp	rs61753987	missense	3.19E-05	25.4	D	D	D	D	H	D	.	.	.	.
c.169T>A	p.Cys57Ser	rs779874391	missense	7.95E-06	25.9	D	D	D	D	M	D	.	.	.	.
c.158T>C	p.Phe53Ser	rs1479532600	missense	3.98E-06	28.1	D	D	D	D	H	D	.	.	.	.
c.154A>T	p.Ser52Cys	rs747701551	missense	3.98E-06	25.7	D	D	D	D	M	D	.	.	.	.
c.8442A>G	p.Ter2814TrpextTer38	rs778161123	stop_lost	4.05E-06	.	.	.	.	.	.	.	.	.	.	.
c.8151delC	p.Asp2717GlufsTer27	rs1264216385	frameshift	3.98E-06	.	.	.	.	.	.	.	.	.	.	.
c.7966_7968delGGA	p.Gly2656del	rs768488894	inframe insdels	3.98E-06	.	.	.	.	.	.	.	.	.	.	.
c.7942delG	p.Ala2648LeufsTer6	rs1429129116	frameshift	3.98E-06	.	.	.	.	.	.	.	.	.	.	.
c.7861A>T	p.Lys2621Ter	rs774563410	stop_gained	7.96E-06	.	.	.	.	.	.	.	.	.	.	.
c.7778_7780delAGA	p.Lys2593del	rs762835479	inframe insdels	3.98E-06	.	.	.	.	.	.	.	.	.	.	.
c.7625delT	p.Ile2542AsnfsTer22	NA	frameshift	3.98E-06	.	.	.	.	.	.	.	.	.	.	.
c.7483delC	p.Leu2495CysfsTer8	rs1229452874	frameshift	7.07E-06	.	.	.	.	.	.	.	.	.	.	.
c.7459G>T	p.Glu2487Ter	rs748315151	stop_gained	3.98E-06	.	.	.	.	.	.	.	.	.	.	.
c.7359C>A	p.Cys2453Ter	rs775194189	stop_gained	3.98E-06	.	.	.	.	.	.	.	.	.	.	.

c.7213_7216dupTGTC	p.Pro2406LeufsTer13	rs752860551	frameshift	3.98E-06	.	.	.	.	.	.	.	.	.	.	.
c.7104_7105delAA	p.Arg2369SerfsTer21	rs1254154100	frameshift	4.00E-06	.	.	.	.	.	.	.	.	.	.	.
c.6940C>T	p.Gln2314Ter	rs1454935692	stop_gained	3.98E-06	.	.	.	.	.	.	.	.	.	.	.
c.6697G>T	p.Glu2233Ter	rs61750623	stop_gained	3.19E-05	.	.	.	.	.	.	.	.	.	.	.
c.6432dupA	p.Pro2145ThrfsTer5	rs61750618	frameshift	3.98E-06	.	.	.	.	.	.	.	.	.	.	.
c.6427_6429delCTC	p.Leu2144del	rs769453980	inframe insdels	3.98E-06	.	.	.	.	.	.	.	.	.	.	.
c.6388dupC	p.Gln2130ProfsTer20	rs1488493898	frameshift	3.98E-06	.	.	.	.	.	.	.	.	.	.	.
c.6320G>A	p.Trp2107Ter	rs377640206	stop_gained	3.98E-06	.	.	.	.	.	.	.	.	.	.	.
c.6188delC	p.Pro2063HisfsTer36	rs1368072612	frameshift	3.98E-06	.	.	.	.	.	.	.	.	.	.	.
c.5992delA	p.Arg1998GlyfsTer5	rs752950172	frameshift	3.98E-06	.	.	.	.	.	.	.	.	.	.	.
c.5943_5948delGCAGGA	p.Glu1981_Gln1982del	rs1303691608	inframe insdels	7.96E-06	.	.	.	.	.	.	.	.	.	.	.
c.5613delG	p.Cys1872AlafsTer31	rs779575058	frameshift	3.98E-06	.	.	.	.	.	.	.	.	.	.	.
c.5535delC	p.Ser1846ProfsTer4	rs1444616480	frameshift	3.98E-06	.	.	.	.	.	.	.	.	.	.	.
c.5469_5474delCCCTAT	p.Pro1824_Ile1825del	rs1477546504	inframe insdels	3.19E-05	.	.	.	.	.	.	.	.	.	.	.
c.5056_5073delTGCAGCCAGCCCCTGGAC	p.Cys1686_Asp1691del	rs548269199	inframe insdels	3.98E-06	.	.	.	.	.	.	.	.	.	.	.
c.4999C>T	p.Gln1667Ter	rs1449653323	stop_gained	4.01E-06	.	.	.	.	.	.	.	.	.	.	.
c.4752C>A	p.Tyr1584Ter	rs1475440343	stop_gained	3.98E-06	.	.	.	.	.	.	.	.	.	.	.
c.4681dupA	p.Ile1561AsnfsTer27	rs777481512	frameshift	3.99E-06	.	.	.	.	.	.	.	.	.	.	.
c.4402_4404delACT	p.Thr1468del	rs1243317800	inframe insdels	4.00E-06	.	.	.	.	.	.	.	.	.	.	.
c.4400delC	p.Pro1467LeufsTer58	NA	frameshift	4.00E-06	.	.	.	.	.	.	.	.	.	.	.
c.4057C>T	p.Gln1353Ter	rs1322770774	stop_gained	3.99E-06	.	.	.	.	.	.	.	.	.	.	.
c.3839_3845dupTCCTGCT	p.Asp1283ProfsTer12	rs61749377	frameshift	3.98E-06	.	.	.	.	.	.	.	.	.	.	.
c.3738_3760delTCCCACAGATGCCCCGGTGAGCC	p.Pro1247HisfsTer38	rs906284277	frameshift	4.04E-06	.	.	.	.	.	.	.	.	.	.	.
c.3713delA	p.Gln1238ArgfsTer15	rs774296321	frameshift	4.09E-06	.	.	.	.	.	.	.	.	.	.	.

c.3270delC	p.Cys1091ValfsTer28	rs775596301	frameshift	4.02E-06	.	.	.	.	.	.	.	.	.	.	.
c.3259_3261delGAC	p.Asp1087del	rs1393068829	inframe insdels	4.02E-06	.	.	.	.	.	.	.	.	.	.	.
c.3252delC	p.Cys1084Ter	rs760804402	frameshift	4.01E-06	.	.	.	.	.	.	.	.	.	.	.
c.3189delT	p.Thr1064ProfsTer55	rs776853073	frameshift	4.57E-06	.	.	.	.	.	.	.	.	.	.	.
c.3102delC	p.Arg1035GlufsTer17	rs146648301	frameshift	3.98E-06	.	.	.	.	.	.	.	.	.	.	.
c.3097_3099dupGAC	p.Asp1033dup	rs752219725	inframe insdels	0.000244955	.	.	.	.	.	.	.	.	.	.	.
c.3011delA	p.Asn1004ThrfsTer24	rs1187947066	frameshift	3.98E-06	.	.	.	.	.	.	.	.	.	.	.
c.2681delT	p.Val894GlyfsTer15	rs748924210	frameshift	3.98E-06	.	.	.	.	.	.	.	.	.	.	.
c.2673C>A	p.Tyr891Ter	rs753317915	stop_gained	3.98E-06	.	.	.	.	.	.	.	.	.	.	.
c.2649C>G	p.Tyr883Ter	rs1171471119	stop_gained	3.98E-06	.	.	.	.	.	.	.	.	.	.	.
c.2582delA	p.His861LeufsTer48	rs758482771	frameshift	3.98E-06	.	.	.	.	.	.	.	.	.	.	.
c.2554C>T	p.Gln852Ter	rs772534075	stop_gained	3.98E-06	.	.	.	.	.	.	.	.	.	.	.
c.2423_2424insTCTCTGGCTT	p.Cys810TrpfsTer12	rs1370519514	frameshift	3.98E-06	.	.	.	.	.	.	.	.	.	.	.
c.2310delC	p.Met771TrpfsTer30	rs1212894308	frameshift	3.98E-06	.	.	.	.	.	.	.	.	.	.	.
c.2146_2148delTTC	p.Phe716del	rs1168682360	inframe insdels	3.18E-05	.	.	.	.	.	.	.	.	.	.	.
c.2067C>A	p.Cys689Ter	rs764755360	stop_gained	7.95E-06	.	.	.	.	.	.	.	.	.	.	.
c.1696dupC	p.His566ProfsTer84	rs745667020	frameshift	4.02E-06	.	.	.	.	.	.	.	.	.	.	.
c.1614delC	p.Ser539LeufsTer38	rs1249758434	frameshift	7.98E-06	.	.	.	.	.	.	.	.	.	.	.
c.1581C>G	p.Tyr527Ter	rs1420962761	stop_gained	4.00E-06	.	.	.	.	.	.	.	.	.	.	.
c.1542dupC	p.Val515ArgfsTer135	rs867631561	frameshift	4.03E-06	.	.	.	.	.	.	.	.	.	.	.
c.1479delC	p.Tyr494ThrfsTer17	rs779876879	frameshift	3.99E-06	.	.	.	.	.	.	.	.	.	.	.
c.1358_1360delACA	p.Asn453del	rs1246069621	inframe insdels	3.98E-06	.	.	.	.	.	.	.	.	.	.	.
c.1329delC	p.Val444SerfsTer13	rs149116506	frameshift	4.00E-06	.	.	.	.	.	.	.	.	.	.	.
c.1147G>T	p.Glu383Ter	rs1227349336	stop_gained	3.18E-05	.	.	.	.	.	.	.	.	.	.	.
c.1025dupG	p.Cys342TrpfsTer41	rs771376802	frameshift	3.98E-06	.	.	.	.	.	.	.	.	.	.	.
c.1026C>A	p.Cys342Ter	rs771376802	stop_gained	3.98E-06	.	.	.	.	.	.	.	.	.	.	.

c.993C>A	p.Cys331Ter	rs147924974	stop_gained	7.96E-06	.	.	.	.	.	.	.	.	.	.	.
c.795_818dupCCCTGCCCTCCTGGAGTACGCCCG	p.Pro266_Arg273dup	rs1006469048	inframe insdels	3.98E-06	.	.	.	.	.	.	.	.	.	.	.
c.793_816dupTGCCCTGCCCTCCTGGAGTACGCC	p.Cys265_Ala272dup	rs61753997	inframe insdels	7.97E-06	.	.	.	.	.	.	.	.	.	.	.
c.781delC	p.Leu261TrpfsTer196	rs1166840721	frameshift	3.98E-06	.	.	.	.	.	.	.	.	.	.	.
c.676C>T	p.Gln226Ter	rs1282064425	stop_gained	4.01E-06	.	.	.	.	.	.	.	.	.	.	.
c.596G>A	p.Trp199Ter	rs1299804437	stop_gained	3.98E-06	.	.	.	.	.	.	.	.	.	.	.
c.439C>T	p.Gln147Ter	rs199939434	stop_gained	3.98E-06	.	.	.	.	.	.	.	.	.	.	.
c.268delG	p.Glu90AsnfsTer13	rs1326486598	frameshift	3.98E-06	.	.	.	.	.	.	.	.	.	.	.
c.8115+4A>G	NA	rs768136976	splicing	7.96E-06	23.3	.	.	.	.	.	.	.	D	?	Splicing effect
c.7986+1G>A	NA	rs1285578671	splicing	3.19E-05	33	.	.	.	.	.	.	pathogenic	D	D	.
c.7887+2T>A	NA	rs113814258	splicing	1.59E-05	33	.	.	.	.	.	.	pathogenic	D	D	.
c.7729+1G>T	NA	rs760277526	splicing	4.07E-06	33	.	.	.	.	.	.	pathogenic	D	D	.
c.7548+1G>A	NA	rs747482652	splicing	7.97E-06	33	.	.	.	.	.	.	pathogenic	?	?	.
c.7287+1G>T	NA	rs1235191685	splicing	3.98E-06	34	.	.	.	.	.	.	pathogenic	D	D	.
c.7081+1G>A	NA	rs112978778	splicing	4.02E-06	33	.	.	.	.	.	.	pathogenic	D	D	.
c.6599-1G>C	NA	rs748810026	splicing	7.98E-06	33	.	.	.	.	.	.	pathogenic	D	D	.
c.5621-2A>C	NA	rs769632868	splicing	3.98E-06	33	.	.	.	.	.	.	pathogenic	D	D	.
c.5621-5T>G	NA	rs1464997603	splicing	6.37E-05	22.2	.	.	.	.	.	.	.	D	D	Likely splicing effect
c.5620+2T>G	NA	rs758879278	splicing	7.96E-06	34	.	.	.	.	.	.	pathogenic	D	D	.
c.5456-2A>T	NA	rs1251888585	splicing	3.98E-06	34	.	.	.	.	.	.	pathogenic	D	D	.
c.5311+1G>A	NA	rs1330344875	splicing	4.00E-06	33	.	.	.	.	.	.	pathogenic	D	?	.
c.5170+1G>A	NA	rs764543553	splicing	3.98E-06	34	.	.	.	.	.	.	pathogenic	D	D	.
c.2968-2A>G	NA	NA	splicing	3.98E-06	34	.	.	.	.	.	.	pathogenic	D	D	.
c.2967+2T>C	NA	rs773737583	splicing	0.000137978	27.1	.	.	.	.	.	.	pathogenic	D	D	.
c.2821-2A>G	NA	rs764126736	splicing	7.96E-06	34	.	.	.	.	.	.	pathogenic	D	D	.

c.2820+2T>G	NA	rs751608635	splicing	3.98E-06	33	.	.	.	.	.	.	pathogenic	D	D	.
c.2686-2A>C	NA	rs61748489	splicing	1.99E-05	33	.	.	.	.	.	.	pathogenic	D	D	.
c.1730-2A>C	NA	rs61754014	splicing	7.50E-06	34	.	.	.	.	.	.	pathogenic	D	D	.
c.1729+1G>A	NA	rs775842350	splicing	8.20E-06	32	.	.	.	.	.	.	pathogenic	?	?	.
c.1294-2A>G	NA	rs1261701961	splicing	4.01E-06	32	.	.	.	.	.	.	pathogenic	D	D	.
c.874+4A>G	NA	rs1228841352	splicing	4.01E-06	23	.	.	.	.	.	.	.	D	D	Splicing effect
c.221-2A>T	NA	rs1195620730	splicing	3.98E-06	35	.	.	.	.	.	.	pathogenic	D	D	.
c.1-3C>G	NA	rs774023147	splicing	3.98E-06	23.8	.	.	.	.	.	.	.	D	D	?

$splice-site variants affecting the first 2 or last 2 intronic

Supplementary Table 2. Identified pathogenic variants in the gnomAD that were reported to be associated with von Willebrand disease (VWD).

Transcript Consequence	Protein Consequence	rsIDs	Type of variant	Allele Frequency	Clinical classification
c.8366C>G	p.Thr2789Ser	rs371036946	missense	3,98E-05	type 2B
c.8347C>T	p.Gln2783Ter	rs1298396777	stop_gained	3,98E-06	type 1
c.8327C>T	p.Pro2776Leu	rs61751312	missense	1,59E-05	type 1
c.8216G>A	p.Cys2739Tyr	rs61751305	missense	7,95E-06	type 3
c.8164C>G	p.Pro2722Ala	rs62641244	missense	4,61E-05	type 1
c.8155+6T>C	NA	rs1223422347	splice,intron	3,98E-06	type 1
c.8148T>A	p.Cys2716Ter	NA	stop_gained	3,98E-06	type 1
c.8084C>G	p.Pro2695Arg	rs76459136	missense	0,000254561	type 1
c.7988G>C	p.Arg2663Pro	rs149834874	splice,missense	0,00142938	type 1
c.7987C>T	p.Arg2663Cys	rs370662678	splice,missense	3,89E-05	type 1
c.7940C>T	p.Thr2647Met	rs61751302	missense	0,003772317	type 1
c.7770+1G>A	NA	rs200770256	splice	3,98E-06	type 1
c.7730-1G>C	NA	rs267607366	splice	3,98E-06	type 3
c.7729+5G>A	NA	rs918057829	splice,intron	1,09E-05	type 1
c.7723C>T	p.Arg2575Cys	rs375991463	missense	4,66E-05	UC
c.7651C>T	p.Gln2551Ter	rs776340794	stop_gained	3,98E-06	type 3
c.7636A>T	p.Asn2546Tyr	rs61751298	missense	3,98E-06	type 3
c.7627C>T	p.Gln2543Ter	rs1368264172	stop_gained	3,98E-06	type 1
c.7604G>A	p.Arg2535Gln	rs137987906	missense	9,55E-05	type 3
c.7603C>T	p.Arg2535Ter	rs61751296	stop_gained	1,77E-05	type 3
c.7558C>T	p.Gln2520Ter	rs1448479214	stop_gained	7,08E-06	type 3
c.7552G>A	p.Gly2518Ser	rs61751293	missense	1,77E-05	type 1
c.7493C>A	p.Ala2498Asp	rs369669154	missense	1,41E-05	UC
c.7489T>C	p.Ser2497Pro	rs61751292	missense	3,98E-06	type 1
c.7437G>A	p.Ser2479Ser	rs267607363	splice,synonymous	2,00E-05	type 3
c.7408C>T	p.Gln2470Ter	rs61751288	stop_gained	1,20E-05	type 1
c.7393G>A	p.Val2465Met	rs375655409	missense	6,37E-05	type 2A
c.7390C>T	p.Arg2464Cys	rs61751286	missense	8,14E-05	type 1
c.7361C>A	p.Thr2454Asn	rs200486416	missense	2,39E-05	type 3
c.7300C>T	p.Arg2434Ter	rs62643640	stop_gained	6,37E-05	type 3
c.7150C>T	p.Arg2384Trp	rs145697622	missense	0,000279672	UC
c.7135C>T	p.Arg2379Cys	rs61751283	missense	3,90E-05	type 1
c.7070A>T	p.Asn2357Ile	rs750296925	missense	1,42E-05	type 2A
c.7028G>T	p.Gly2343Val	rs61750629	missense	3,99E-06	type 1
c.7025G>A	p.Arg2342His	rs34120165	missense	0,000382862	UC
c.6989T>G	p.Val2330Gly	rs761604234	missense	3,99E-06	type 1
c.6973T>A	p.Cys2325Ser	rs1256082707	missense	3,98E-06	type 3
c.6938G>A	p.Arg2313His	rs62641242	missense	0,000321712	type 1
c.6932G>A	p.Arg2311His	rs267607357	missense	1,99E-05	type 1
c.6890C>T	p.Pro2297Leu	rs201372397	missense	3,99E-05	UC
c.6860G>A	p.Arg2287Gln	rs563856279	missense	0,000424836	UC
c.6859C>T	p.Arg2287Trp	rs61750625	missense	0,000761175	type 1
c.6798+1G>T	NA	rs61750624	splice	3,98E-06	type 1
c.6709T>C	p.Cys2237Arg	rs770625592	missense	1,59E-05	type 3
c.6554G>A	p.Arg2185Gln	rs2229446	missense	0,019553588	type 1
c.6553C>T	p.Arg2185Trp	rs569962285	missense	3,59E-05	type 1
c.6433C>T	p.Pro2145Ser	rs61750618	missense	0,000127305	type 1

c.6433C>G	p.Pro2145Ala	rs61750618	missense	1,59E-05	type 2M
c.6352C>T	p.Arg2118Trp	rs200719767	missense	4,95E-05	type 1
c.6311C>T	p.Thr2104Ile	rs61750616	missense	7,97E-06	type 1
c.6187C>T	p.Pro2063Ser	rs61750615	missense	0,011720987	type 3
c.6104G>A	p.Gly2035Asp	rs186806674	missense	0,000403363	type 1
c.6068C>T	p.Thr2023Met	rs761576269	missense	3,90E-05	type 2M
c.5851A>G	p.Thr1951Ala	rs144072210	missense	0,000653191	type 1
c.5801T>G	p.Val1934Gly	rs139845585	missense	1,07E-05	type 1
c.5793G>C	p.Gln1931His	rs574811308	missense	0,000321347	type 3
c.5695T>C	p.Cys1899Arg	rs559785610	missense	4,14E-06	type 1
c.5692C>T	p.Gln1898Ter	rs749765999	stop_gained	4,18E-06	type 3
c.5557C>T	p.Arg1853Ter	rs61750612	stop_gained	1,59E-05	type 3
c.5545G>A	p.Val1849Met	rs61750611	missense	7,95E-06	type 1
c.5509C>T	p.Arg1837Trp	rs1250634491	missense	1,19E-05	type 1
c.5453A>G	p.Asn1818Ser	rs61750608	splice,missense	7,16E-05	type 1
c.5347T>G	p.Ser1783Ala	rs267607353	missense	0,000143145	type 2M
c.5335C>T	p.Arg1779Ter	rs61750606	stop_gained	7,95E-06	type 1
c.5312-2_5312-1delAG	NA	rs1287088175	splice	3,18E-05	type 1
c.5311G>A	p.Gly1771Arg	rs370016586	missense	1,20E-05	type 3
c.5282T>A	p.Met1761Lys	rs1490774216	missense	1,19E-05	type 2M
c.5278G>A	p.Val1760Ile	rs61750604	missense	0,000756777	type 1
c.5200C>T	p.Gln1734Ter	rs374707563	stop_gained	1,06E-05	type 3
c.5191T>A	p.Ser1731Thr	rs61750603	missense	0,001456396	type 2M
c.5180_5181insTT	p.Thr1728SerfsTer29	rs61750602	frameshift	3,98E-06	type 1
c.5014G>A	p.Gly1672Arg	rs61750598	missense	0,000383378	type 2A
c.5004G>T	p.Arg1668Ser	rs61750597	missense	2,81E-05	type 1
c.4975C>T	p.Arg1659Ter	rs61750595	stop_gained	5,68E-05	type 3
c.4944delT	p.Ile1649SerfsTer44	rs1306900762	frameshift	1,99E-05	type 1
c.4904A>T	p.Asn1635Ile	rs778661133	missense	1,59E-05	type 2A
c.4850A>G	p.Lys1617Arg	rs559911446	missense	1,77E-05	type 1
c.4840G>A	p.Asp1614Asn	rs1478977315	missense	3,98E-06	type 2A
c.4825G>A	p.Gly1609Arg	rs61750580	missense	3,98E-06	type 2A
c.4751A>G	p.Tyr1584Cys	rs1800386	missense	0,002630219	type 1
c.4747C>T	p.Arg1583Trp	rs61750116	missense	3,89E-05	UC
c.4717G>A	p.Gly1573Ser	rs267607349	missense	3,99E-06	type 2A
c.4696C>T	p.Arg1566Ter	rs61750112	stop_gained	7,98E-06	type 1
c.4690C>T	p.Arg1564Trp	rs370854023	missense	1,20E-05	type 1
c.4636delG	p.Val1546Ter	rs1329702479	frameshift	7,98E-06	type 3
c.4580G>A	p.Arg1527Gln	rs780538558	missense	7,80E-05	type 2A
c.4552A>G	p.Lys1518Glu	rs61750102	missense	3,98E-06	type 2A
c.4492G>A	p.Asp1498Asn	rs1009808532	missense	1,20E-05	type 2A
c.4414_4415insC	p.Asp1472AlafsTer40	rs267607339	frameshift	3,19E-05	type 3
c.4384C>G	p.Pro1462Ala	rs61750090	missense	1,20E-05	UC
c.4378C>T	p.Leu1460Phe	rs61750088	missense	1,60E-05	type 2B
c.4339G>C	p.Glu1447Gln	rs61750085	missense	1,19E-05	type 1
c.4315G>A	p.Val1439Met	rs150077670	missense	0,001001777	type 2M
c.4309G>A	p.Ala1437Thr	rs61750084	missense	7,96E-06	type 2M
c.4276C>T	p.Arg1426Cys	rs555366738	missense	1,19E-05	type 2M
c.4273A>T	p.Ile1425Phe	rs61750083	missense	3,98E-06	type 2M
c.4255C>A	p.His1419Asn	rs375498783	missense	5,66E-05	type 2A
c.4247T>C	p.Ile1416Thr	rs61750081	missense	3,98E-06	type 2M

c.4238C>T	p.Pro1413Leu	rs61750079	missense	1,06E-05	type 1
c.4195C>T	p.Arg1399Cys	rs61750077	missense	2,84E-05	type 2M
c.4183C>T	p.Arg1395Trp	rs751394243	missense	1,42E-05	type 2M
c.4181C>T	p.Ser1394Phe	rs1033516182	missense	1,42E-05	type 2M
c.4176delG	p.Met1393CysfsTer12	rs781101010	frameshift	4,00E-06	type 3
c.4162C>T	p.Gln1388Ter	rs1205530944	stop_gained	4,01E-06	type 1
c.4135C>T	p.Arg1379Cys	rs61750074	missense	2,13E-05	type 2B
c.4120C>T	p.Arg1374Cys	rs61750071	missense	3,98E-06	type 2M
c.4117G>T	p.Asp1373Tyr	rs1332206266	missense	3,98E-06	type 2M
c.4115T>G	p.Ile1372Ser	rs61750070	missense	3,19E-05	type 2B
c.4079T>C	p.Val1360Ala	rs267607338	missense	3,19E-05	type 2M
c.4075G>A	p.Glu1359Lys	rs61749407	missense	1,42E-05	type 2M
c.4027A>G	p.Ile1343Val	rs150923481	missense	0,000106338	type 3
c.4024C>T	p.Arg1342Cys	rs61749404	missense	0,000116996	type 1
c.4022G>A	p.Arg1341Gln	rs61749403	missense	3,99E-06	type 2B
c.4021C>T	p.Arg1341Trp	rs61749402	missense	2,39E-05	type 2B
c.4010C>T	p.Pro1337Leu	rs61749400	missense	3,99E-06	type 2B
c.4001G>A	p.Arg1334Gln	rs775812331	missense	7,99E-06	type 2M
c.4000C>T	p.Arg1334Trp	rs746810319	missense	2,84E-05	type 2M
c.3974C>T	p.Ser1325Phe	rs538488005	missense	3,99E-06	type 2M
c.3970G>A	p.Gly1324Ser	rs61749398	missense	7,98E-06	type 2M
c.3944G>A	p.Arg1315His	rs61749396	missense	2,39E-05	type 1
c.3931C>T	p.Gln1311Ter	rs267607337	stop_gained	5,18E-05	type 3
c.3923G>T	p.Arg1308Leu	rs61749388	missense	3,98E-06	type 2B
c.3923G>A	p.Arg1308His	rs61749388	missense	4,60E-05	type 2A
c.3897delT	p.Phe1299LeufsTer5	rs754172743	frameshift	3,98E-06	type 3
c.3835G>A	p.Val1279Ile	rs61749376	missense	0,000294999	type 2B
c.3797C>T	p.Pro1266Leu	rs61749370	missense	0,000832207	type 2B
c.3797C>A	p.Pro1266Gln	rs61749370	missense	0,000270289	type 2B
c.3773A>G	p.Tyr1258Cys	rs781111573	missense	5,22E-05	type 2B
c.3692A>G	p.Asn1231Ser	rs61749368	missense	0,001291189	type 1
c.3692A>C	p.Asn1231Thr	rs61749368	missense	0,001674168	type 1
c.3679T>C	p.Cys1227Arg	rs61749366	missense	4,17E-06	type 1
c.3675-1G>A	NA	rs746457842	splice	4,20E-06	type 3
c.3613C>T	p.Arg1205Cys	rs373787920	missense	1,19E-05	type 1
c.3586T>C	p.Cys1196Arg	rs61749365	missense	3,98E-06	UC
c.3583G>T	p.Asp1195Tyr	rs374591991	missense	1,06E-05	type 2A
c.3379+1G>A	NA	rs2363337	splice	2,49E-05	type 1
c.3281T>C	p.Ile1094Thr	rs267607317	missense	8,95E-05	type 1
c.3159G>T	p.Gln1053His	rs61748496	missense	2,37E-05	type 2N
c.3108+5G>A	NA	rs61748495	splice,intron	3,98E-06	type 3
c.3101_3103delCCA	p.Thr1034del	rs368366214	inframe_deletion	0,001543002	type 3
c.2989G>A	p.Gly997Arg	NA	missense	3,18E-05	UC
c.2944G>C	p.Val982Leu	rs376548659	missense	2,48E-05	type 3
c.2926C>T	p.Arg976Cys	rs764251476	missense	3,98E-05	type 2A
c.2900G>T	p.Gly967Val	rs141087261	missense	7,96E-06	type 3
c.2900G>A	p.Gly967Asp	rs141087261	missense	0,002514607	UC
c.2878C>T	p.Arg960Trp	rs370984712	missense	0,000139229	type 1
c.2820+5G>C	NA	rs569571599	splice,intron	3,98E-06	type 3
c.2771G>A	p.Arg924Gln	rs33978901	missense	0,010665752	type 1
c.2770C>T	p.Arg924Trp	rs61748491	missense	2,78E-05	type 1

c.2686-2A>G	NA	rs61748489	splice	3,98E-06	type 1
c.2635G>A	p.Asp879Asn	rs61748485	missense	2,78E-05	type 2N
c.2574C>G	p.Cys858Trp	rs184227165	missense	7,07E-06	type 1
c.2561G>A	p.Arg854Gln	rs41276738	missense	0,003465052	type 2N
c.2560C>T	p.Arg854Trp	rs61748482	missense	1,99E-05	type 2N
c.2546G>T	p.Cys849Phe	rs772796741	missense	7,95E-06	type 2N
c.2516delG	p.Gly839GlufsTer4	rs1347159253	frameshift	1,06E-05	type 1
c.2451T>A	p.His817Gln	rs57950734	missense	0,011218912	type 2N
c.2447G>A	p.Arg816Gln	rs62643634	missense	7,07E-06	type 2N
c.2446C>T	p.Arg816Trp	rs121964894	missense	6,01E-05	type 2N
c.2443-1G>C	NA	rs61748480	splice	3,98E-06	type 3
c.2438dupG	p.Met814HisfsTer5	rs757149909	frameshift	7,97E-06	type 1
c.2435delC	p.Pro812ArgfsTer31	rs745322229	frameshift	0,000116911	type 3
c.2435C>T	p.Pro812Leu	rs62643631	missense	0,000103643	type 2N
c.2372C>T	p.Thr791Met	rs61748477	missense	1,06E-05	type 2N
c.2359G>A	p.Glu787Lys	rs61748474	missense	3,98E-06	type 2N
c.2345G>A	p.Arg782Gln	rs61748472	missense	1,77E-05	type 1
c.2344C>T	p.Arg782Trp	rs61748471	missense	5,66E-05	type 2N
c.2303G>A	p.Arg768Gln	rs772203447	missense	5,31E-05	type 1
c.2279G>A	p.Arg760His	rs61748467	splice,missense	1,77E-05	type 2A
c.2278C>T	p.Arg760Cys	rs61748466	missense	1,77E-05	type 2N
c.2269_2270delCT	p.Leu757ValfsTer22	rs61748465	frameshift	7,96E-06	type 3
c.2220G>A	p.Met740Ile	rs2228317	missense	0,018042586	UC
c.2072delC	p.Pro691GlnfsTer50	rs1258485397	frameshift	3,98E-06	type 1
c.2016_2019delCTCT	p.Ser673ThrfsTer67	rs371126726	frameshift	3,18E-05	type 3
c.1946-4C>T	NA	rs61748461	splice,intron	1,32E-05	type 3
c.1922C>T	p.Ala641Val	rs61754019	missense	0,0011256	type 1
c.1892C>T	p.Ala631Val	rs199963222	missense	0,000316546	type 1
c.1870G>A	p.Gly624Ser	rs542226383	missense	0,000375061	type 2A
c.1847C>T	p.Ser616Leu	rs1225435169	missense	2,20E-05	UC
c.1781C>G	p.Ala594Gly	rs267607308	missense	9,81E-05	type 1
c.1730-5C>T	NA	rs569984866	splice,intron	0,000679756	type 3
c.1728G>T	p.Met576Ile	rs150146744	splice,missense	0,004972612	type 1
c.1657dupT	p.Trp553LeufsTer97	rs267607307	frameshift	3,19E-05	type 3
c.1654G>A	p.Ala552Thr	rs779932077	missense	1,20E-05	type 2N
c.1583A>G	p.Asn528Ser	rs61754010	missense	4,00E-06	type 2A
c.1534-3C>A	NA	rs61754009	splice	7,17E-06	type 1
c.1497G>C	p.Gln499His	rs774725519	missense	4,00E-06	type 1
c.1446C>G	p.Ile482Met	rs569669757	missense	0,000199998	type 2A
c.1339C>T	p.Arg447Trp	rs372495746	missense	3,19E-05	type 3
c.1309G>T	p.Asp437Tyr	rs375486035	missense	0,000112153	type 3
c.1239dupG	p.Leu414AlafsTer15	rs375451251	frameshift	7,08E-06	type 3
c.1135T>G	p.Cys379Gly	rs763461692	missense	7,96E-06	type 1
c.1117C>T	p.Arg373Ter	rs62643625	stop_gained	2,39E-05	type 3
c.1109G>A	p.Cys370Tyr	rs763827767	missense	3,98E-06	UC
c.1001G>A	p.Gly334Glu	rs932134873	missense	3,99E-06	type 2N
c.971G>A	p.Arg324Gln	rs61754001	missense	2,78E-05	type 1
c.970C>T	p.Arg324Ter	rs61754000	stop_gained	1,19E-05	type 3
c.962G>A	p.Cys321Tyr	rs766016814	missense	2,83E-05	type 1
c.871delT	p.Cys291AlafsTer166	NA	frameshift	4,00E-06	type 3
c.817C>T	p.Arg273Trp	rs61753997	missense	2,79E-05	type 3

c.813C>G	p.Tyr271Ter	rs750697933	stop_gained	7,97E-06	type 3
c.780dupG	p.Leu261AlafsTer42	rs760130928	frameshift	7,97E-06	type 3
c.760_761delTT	p.Leu254ValfsTer2	rs1435596998	frameshift	7,96E-06	type 3
c.658-3C>A	NA	rs377196768	splice,intron	3,99E-05	type 3
c.646G>A	p.Glu216Lys	rs767837153	missense	9,55E-05	type 1
c.605G>C	p.Arg202Pro	rs369737556	missense	1,19E-05	type 2A
c.605G>A	p.Arg202Gln	rs369737556	missense	1,19E-05	type 2A
c.604C>T	p.Arg202Trp	rs990682639	missense	1,06E-05	type 2A
c.514G>A	p.Asp172Asn	rs766305860	missense	3,98E-06	type 2A
c.469A>G	p.Lys157Glu	rs553810662	missense	7,07E-06	type 3
c.449T>C	p.Leu150Pro	rs61753994	missense	3,98E-06	type 3
c.414_426delCAGGATC GATGGC	p.Arg139AlafsTer32	rs1312486904	frameshift	3,98E-06	type 3
c.385C>A	p.Leu129Met	rs61753991	missense	0,000752953	type 1
c.260A>C	p.Tyr87Ser	rs62643621	missense	1,06E-05	UC
c.250C>T	p.Leu84Phe	rs372664002	missense	0,000152024	type 2A
c.115G>A	p.Gly39Arg	rs1397778191	missense	7,08E-06	type 1
c.100C>T	p.Arg34Ter	rs61753984	stop_gained	1,19E-05	type 3
c.50dupT	p.Leu17PhefsTer25	rs751286556	frameshift	1,41E-05	type 3

UC: unclassified variant

Supplementary Table 3. Gene constraint of *VWF*.

**Type of variant**	**Expected no. of variants**	**Observed no. of variants**	**Constraint metric**
Synoymous	666.3	728	Z= -1.88<br>o/e= 1.09 (1.03-1.16)
Missense	1602.5	1491	Z= 0.99<br>o/e= 0.93 (0.89-0.97)
pLoF	133	64	pLI= 0<br>o/e= 0.48 (0.39-0.59)

Supplementary Table 4. Type of genetic variants in 1026 individuals who were homozygous for *VWF* pathogenic variants.

Variant	Protein Consequence	Type of variant	Homozygote Count	Ethnicity (n. of homozygous cases/total n. of cases)	Reported type of VWD variant
c.3101_3103delCCA	p.Thr1034del	Inframe deletion	3	African/African American (3/12487)	Type 3
c.3097_3099dupGAC	p.Asp1033dup	Inframe insertion	1	Latino/Admixed American (1/17720)	Type 3
c.6187C>T	p.Pro2063Ser	missense	67	Latino/Admixed American (2/17720), Ashkenazi Jewish (7/5185), non-Finnish European (10/64603), South Asian (46/15308), Other (2/3614)	Type 1
c.7940C>T	p.Thr2647Met	missense	6	Finnish (4/12562), non-Finnish European (2/64603)	Type 1
c.7025G>A	p.Arg2342His	missense	1	Ashkenazi Jewish (1/5185)	UC
c.6859C>T	p.Arg2287Trp	missense	1	African/African American (1/12487)	Type 1
c.6554G>A	p.Arg2185Gln	missense	472	African/African American (465/12487)*	Type 1
c.6104G>A	p.Gly2035Asp	missense	2	East Asian (2/9977)	Type 1
c.5793G>C	p.Gln1931His	missense	2	South Asian (2/15308)	Type 3
c.5278G>A	p.Val1760Ile	missense	1	non-Finnish European (1/64603)	Type 1
c.5191T>A	p.Ser1731Thr	missense	1	Ashkenazi Jewish (1/5,185)	2MCB
c.4751A>G	p.Tyr1584Cys	missense	4	European (non-Finnish (4/64603)	Type 1
c.3797C>T	p.Pro1266Leu	missense	1	Finnish European (1/12562)	Type 2B NY
c.3797C>A	p.Pro1266Gln	missense	2	South Asian (2/15308)	Type 2B NY
c.3692A>G	p.Asn1231Ser	missense	5	South Asian (5/15308)	Type 2B NY
c.3692A>C	p.Asn1231Thr	missense	2	South Asian (2/15308)	Type 2B NY
c.2900G>A	p.Gly967Asp	missense	15	African/African American (15/12487)	UC
c.2771G>A	p.Arg924Gln	missense	33	non-Finnish European (30/64603), Latino/Admixed American (1/17720)), South Asian (1/15308), Other (1/3614)	Type 1
c.2561G>A	p.Arg854Gln	missense	5	non-Finnish (3/64603), Finnish European (2/12562)	Type 2N
c.2451T>A	p.His817Gln	missense	152	African/African American (152/12487)	Type 2N
c.2220G>A	p.Met740Ile	missense	396	African/African American (391/12487), Latino/Admixed American (3/17720), non-Finnish European (2/64603)	UC
c.1870G>A	p.Gly624Ser	missense	1	East Asian (1/9977)	Type 2A
c.1728G>T	p.Met576Ile	missense	32	Latino/Admixed American (32/17720)	Type 1
c.385C>A	p.Leu129Met	missense	1	African/African American (1/12487)	Type 1

UC, unclassified variant; * remaining count: Latino/Admixed American (n= 1), Ashkenazi Jewish (n= 1), non-Finnish European (n= 1), South Asian (n= 2) and other (n=2).

Supplementary Table 5. Estimated global prevalence of autosomal dominant von Willebrand disease (VWD) type 1.

Population	Total number of alleles	Total number of affected alleles by type 1 variants	Collective frequency of affected alleles	Heterozygote frequency	Type 1 VWD prevalence in 1000 individuals (autosomal dominant), n variant (n=78)
**All ‡**	257938	9914	0.04	0.07	73.9
**Latino/Admixed American**	35440	2107	0.06	0.11	111.8
**Ashkenazi Jewish**	10370	155	0.01	0.03	29.4
**East Asian**	19954	208	0.01	0.02	20.6
**Finnish**	25124	785	0.03	0.06	60.5
**European (not Finnish)**	129206	4767	0.04	0.07	71.1
**South Asian**	30616	756	0.02	0.05	48.2
**Other ethnicities**	7228	292	0.04	0.08	77.5
**African/African American §**	24974	844	0.03	0.07	65.3

‡ The global prevalence of VWD type 1 is calculated after excluding the common genetic variant p.Arg2185Gln identified in the African/American ethnicity. § After excluding p.Arg2185Gln variant.

Supplementary Table 6. Estimated global prevalence of autosomal dominant von Willebrand disease (VWD) type 2A.

Population	Total number of alleles	Total number of affected alleles by type 2A variants	Collective frequency of affected alleles	Heterozygote frequency	Type 2A VWD prevalence in 1000 individuals (autosomal dominant), n variant (n=23)
**All**	257938	374	0.0014	0.0029	2.9
**Latino/Admixed American**	35440	79	0.0022	0.0044	4.4
**Ashkenazi Jewish**	10370	2	0.0002	0.0004	0.4
**East Asian**	19954	104	0.0052	0.0104	10.4
**Finnish**	25124	19	0.0008	0.0015	1.5
**European (not Finnish)**	129206	128	0.0010	0.0020	2.0
**South Asian**	30616	15	0.0005	0.0010	1.0
**Other ethnicities**	7228	13	0.0018	0.0036	3.6
**African/African American**	24974	14	0.0006	0.0011	1.1

Supplementary Table 7. Estimated global prevalence of autosomal dominant von Willebrand disease (VWD) type 2B.

Population	Total number of alleles	Total number of affected alleles by type 2B variants	Collective frequency of affected alleles	Heterozygote frequency	Type 2B VWD prevalence in 1000 individuals (autosomal dominant), n variant (n=12)
**All**	257938	434	0.0017	0.0034	3.4
**Latino/Admixed American**	35440	66	0.0019	0.0037	3.7
**Ashkenazi Jewish**	10370	55	0.0053	0.0106	10.6
**East Asian**	19954	2	0.0001	0.0002	0.2
**Finnish**	25124	92	0.0037	0.0073	7.3
**European (not Finnish)**	129206	135	0.0010	0.0021	2.1
**South Asian**	30616	60	0.0020	0.0039	3.9
**Other ethnicities**	7228	14	0.0019	0.0039	3.9
**African/African American**	24974	10	0.0004	0.0008	0.8

Supplementary Table 8. Estimated global prevalence of autosomal dominant von Willebrand disease (VWD) type 2M.

Population	Total number of alleles	Total number of affected alleles by type 2M variants	Collective frequency of affected alleles	Heterozygote frequency	Type 2M VWD prevalence in 1000 individuals (autosomal dominant), n variant (n=21)
**All**	257938	792	0.0031	0.0061	6.1
**Latino/Admixed American**	35440	46	0.0013	0.0026	2.6
**Ashkenazi Jewish**	10370	220	0.0212	0.0415	41.5
**East Asian**	19954	8	0.0004	0.0008	0.8
**Finnish**	25124	124	0.0049	0.0098	9.8
**European (not Finnish)**	129206	181	0.0014	0.0028	2.8
**South Asian**	30616	71	0.0023	0.0046	4.6
**Other ethnicities**	7228	24	0.0033	0.0066	6.6
**African/African American**	24974	118	0.0047	0.0094	9.4

Supplementary Table 9. Estimated global prevalence of autosomal recessive von Willebrand disease (VWD) type 2N.

Population	Total number of alleles	Total number of affected alleles by types 1_3/type 2N variants	Collective frequency of affected alleles	Heterozygote frequency	Type 2N VWD prevalence in 1000 individuals (autosomal recessive), n variant (n=143)*	Type 2N VWD carrier prevalence in 1000 individuals, n variant (n=143)
**All**	257938	8064	0.03	0.0606	0.98	61
**Latino/Admixed American**	35440	1705	0.05	0.0916	2.31	92
**Ashkenazi Jewish**	10370	85	0.01	0.0163	0.07	16
**East Asian**	19954	213	0.01067	0.0211	0.11	21
**Finnish**	25124	809	0.03	0.0623	1.04	62
**European (not Finnish)**	129206	3040	0.02	0.0459	0.55	46
**South Asian**	30616	830	0.03	0.0528	0.73	53
**Other ethnicities**	7228	187	0.03	0.0504	0.67	50
**African/African American**	24974	1195	0.05	0.091	2.29	91

*The global prevalence of VWD type 2N was calculated using type 1 or 3 variants with type 2N variants, after removing all variants with a MAF> 1%.

Supplementary Table 10. Estimated global prevalence of autosomal recessive von Willebrand disease (VWD) type 3.

Population	Total number of alleles	Total number of affected alleles by type1 and/or type 3 variants	Collective frequency of affected alleles	Heterozygote frequency	Type 3 VWD prevalence in 1000 individuals (autosomal recessive), n variant (n=129)*	Type 3 VWD carrier prevalence in 1000 individuals, n variant (n=129)
**All**	257938	6991	0.0271	0.053	0.7	5
**Latino/Admixed American**	35440	1610	0.0454	0.087	2.1	9
**Ashkenazi Jewish**	10370	75	0.0072	0.014	0.1	1
**East Asian**	19954	212	0.0106	0.021	0.1	2
**Finnish**	25124	665	0.0265	0.052	0.7	5
**European (not Finnish)**	129206	2328	0.0180	0.035	0.3	4
**South Asian**	30616	782	0.0255	0.050	0.7	5
**Other ethnicities**	7228	161	0.0223	0.044	0.5	4
**African/African American**	24974	1158	0.0464	0.0880	2.2	9

* The global prevalence of VWD type 3 was calculated using both type 1 and type 3 (n= 54) variants, after removing all variants with a MAF> 1%. s
